# Sunflower resistance to multiple downy mildew pathotypes revealed by recognition of conserved effectors of the oomycete *Plasmopara halstedii*

**DOI:** 10.1111/tpj.14157

**Published:** 2019-01-07

**Authors:** Yann Pecrix, Luis Buendia, Charlotte Penouilh‐Suzette, Maude Maréchaux, Ludovic Legrand, Olivier Bouchez, David Rengel, Jérôme Gouzy, Ludovic Cottret, Felicity Vear, Laurence Godiard

**Affiliations:** ^1^ LIPM Laboratoire des Interactions Plantes‐Microorganismes Université de Toulouse INRA CNRS F‐31326 Castanet‐Tolosan France; ^2^ GeT‐PlaGe US INRA 1426 INRA Auzeville F‐31326 Castanet‐Tolosan Cedex France; ^3^ INRA UMR 1095 F‐63000 Clermont‐Ferrand France

**Keywords:** broad‐spectrum resistance, *Helianthus annuus* (cultivated sunflower), pathogen effector network, oomycete, pattern‐triggered immunity suppression, physical mapping of sunflower resistance gene, *Plasmopara halstedii*, RXLR effector, subcellular localization

## Abstract

Over the last 40 years, new sunflower downy mildew isolates (*Plasmopara halstedii*) have overcome major gene resistances in sunflower, requiring the identification of additional and possibly more durable broad‐spectrum resistances. Here, 354 RXLR effectors defined *in silico* from our new genomic data were classified in a network of 40 connected components sharing conserved protein domains. Among 205 RXLR effector genes encoding conserved proteins in 17 *P. halstedii* pathotypes of varying virulence, we selected 30 effectors that were expressed during plant infection as potentially essential genes to target broad‐spectrum resistance in sunflower. The transient expression of the 30 core effectors in sunflower and in *Nicotiana benthamiana* leaves revealed a wide diversity of targeted subcellular compartments, including organelles not so far shown to be targeted by oomycete effectors such as chloroplasts and processing bodies. More than half of the 30 core effectors were able to suppress pattern‐triggered immunity in *N. benthamiana*, and five of these induced hypersensitive responses (HR) in sunflower broad‐spectrum resistant lines. HR triggered by *PhRXLRC01* co‐segregated with *Pl22* resistance in F3 populations and both traits localized in 1.7 Mb on chromosome 13 of the sunflower genome. *Pl22* resistance was physically mapped on the sunflower genome recently sequenced, unlike all the other downy mildew resistances published so far. *PhRXLRC01* and *Pl22* are proposed as an avirulence/resistance gene couple not previously described in sunflower. Core effector recognition is a successful strategy to accelerate broad‐spectrum resistance gene identification in complex crop genomes such as sunflower.

## Significance Statement

Five RXLR‐type effectors of the sunflower downy mildew pathogen conserved in 17 pathotypes of varying virulence, triggered plant immunity in six broad‐spectrum resistant sunflower lines, including two unpublished resistances to all pathotypes. Effector recognition accelerated plant resistance gene identification and fine mapping in complex crop genomes such as sunflower, for which no resistance gene has so far been characterized.

## Introduction

Downy mildew caused by *Plasmopara halstedii* is a major disease affecting sunflower (*Helianthus annuus* L.), the fourth most important oil crop in the world after oil palm, soybean and rapeseed. *P. halstedii* is an obligate biotrophic oomycete, requiring a living *Helianthus* host to complete its life cycle and still has not been successfully cultured *in vitro*. It belongs to the Peronosporales, a devastating oomycetes order including the hemibiotroph genus *Phytophthora*, which causes late blight, and obligate biotrophs, which cause downy mildews (Fawke *et al*., 2015). In spring, *P. halstedii* infects sunflower seedlings through the roots, leading eventually to plant death. It colonizes the hypocotyl, cotyledons and leaves where sporulation and disease symptoms are observed, seriously affecting yield (Gascuel *et al*., 2015). Worldwide, 36 pathotypes of *P*. *halstedii* have so far been identified, of which 16 are known in France (Gascuel *et al*., 2015). Pathotypes are defined by an international nomenclature system based on differential virulence profiles on a set of sunflower inbred lines containing different resistance (*R*) genes called *Pl* (Gascuel *et al*., 2015). More than 20 *Pl* genes conferring resistance to at least one pathotype of *P. halstedii* have been described and mapped on five chromosomes (1, 2, 4, 8 and 13) (Gascuel *et al*., 2015; Qi *et al*., 2015, 2016; Zhang *et al*., 2017) in clusters showing duplications (Bachlava *et al*., 2011) and many resistance gene candidates (Radwan *et al*., 2003, 2008). Attempts to clone these (Slabaugh *et al*., 2003; Franchel *et al*., 2013; Ma *et al*., 2017) have been hampered by reduced recombination frequencies in introgressed regions containing *Pl* genes, high content of repetitive DNA regions in the sunflower genome (Badouin *et al*., 2017), and by the difficulty of transforming sunflower to validate candidate genes (Burrus *et al*., 1996). During the last 40 years, several resistance genes used in sunflower cultivars have become ineffective against new *P*. *halstedii* pathotypes (Ahmed *et al*., 2012). Characterization of further resistances effective against such pathotypes is thus an agronomic issue.

Plant‐pathogenic oomycetes rely for their developmental cycle on pathogenicity factors, called effectors, that are proteins secreted by the pathogen that can alter host defense reactions and advance the infection process (Fawke *et al*., 2015). Cytoplasmic RXLR and CRN effectors are secreted by many oomycetes from infection/nutrition invaginations called haustoria and translocated into host cells by specific amino acid sequences RXLR‐EER or LxLFLAK, respectively (Whisson *et al*., 2007; Schornack *et al*., 2010). During sunflower infection, *P. halstedii* shows intercellular growth, produces haustoria in plant cells and expresses RXLR and CRN effectors (As‐sadi *et al*., 2011; Gascuel *et al*., 2015, 2016a; Sharma *et al*., 2015; Mestre *et al*., 2016). Transient overexpression of *P. halstedii* RXLR and CRN in sunflower leaves triggered HR‐like responses in some resistant lines (Gascuel *et al*., 2016b).

Effectoromics, a high‐throughput functional genomics approach that uses effectors to probe plant germplasm (Vleeshouwers *et al*., 2008; Pais *et al*., 2013) was used to identify sunflower *R* genes to downy mildew. We performed *in silico* genome‐wide identification of *P. halstedii* RXLR effectors from a new genome assembly and analysed them in an interactive network based on conserved protein domains. This network on which were mapped expression and diversity data was a decision‐making tool to select 30 ‘core’ effectors, conserved at the protein level in 17 *P. halstedii* pathotypes. Transient expression experiments of the 30 effectors in sunflower and in *N. benthamiana* revealed a wide diversity of targeted subcellular compartments. These effectors were tested for their ability to trigger HR in sunflower lines carrying 13 *Pl R* genes and two uncharacterized resistance sources. Five core effectors triggered HR in six sunflower lines being each resistant to several downy mildew pathotypes. HR induced by the core effector PhRXLR‐C01 co‐segregated genetically with the *Pl22* resistance gene and these traits were mapped, on the sunflower genome (Badouin *et al*., 2017). The core effector *PhRXLR‐C01* and the *R* gene *Pl22* conferring resistance to nine *P. halstedii* pathotypes are proposed as an AVR/R couple not previously identified in sunflower.

## Results

### Genome assembly of the *P. halstedii* pathotype 710

#### Genome assembly features

We generated and annotated a genome assembly of *P. halstedii* pathotype 710 (Figure S1, Table S1, see Methods). Metrics comparisons with the published *P. halstedii* assembly (strain OS‐Ph8‐99‐BlA4, Sharma *et al*., 2015) suggest that our assembly is less fragmented, as it is composed of less scaffolds (745 versus 3162), less contigs (devoid of ‘N’) (3051 versus 7857) with a larger N50 for our genomic contigs (56.4 kb versus 16.2 kb). Our final assembly has a total length of 67.6 Mb. Its completeness was assessed using BUSCO 3.0.2 (Waterhouse *et al*., 2018). This indicated the presence of 87.2% of the conserved genes from the Stramenopiles and Alveolata database at the genome level, and 88.9% at the proteome level, similar to the earlier published proteome (Table S2). We predicted from our genome assembly 36 892 protein‐coding genes (minimum length of the coding sequence of 40 amino acids). A reciprocal best hit Blast search found 10 608 proteins in common with the previous genome (15 469 predicted proteins) (Table S3).

#### Comparative analysis of *P. halstedii 710 and OS‐Ph8‐99‐BlA4 genome assemblies*

To assess the accuracy of our genome assembly, we compared it to genome assemblies of the closest sequenced oomycete *Plasmopara viticola,* the grapevine downy mildew (Dussert *et al*., 2016, 2018; Mestre *et al*., 2016; Yin *et al*., 2017). We did the same comparisons with the genome published by Sharma *et al*. (2015) (Figure S2). The total length of regions showing similarity between *P*. *halstedii* and *P. viticola* spanned 8–11 Mb, depended on the *P. viticola* genome assembly used. These related regions were carried by less scaffolds on our assembly than on that of Sharma *et al*. (2015) (ratio from 0.47 to 0.61; Figure S2a), this was also illustrated by their distribution in violin plots (Figure S2b). The circos plots (Krzywinski *et al*., 2009) showing syntenic regions between the three largest scaffolds of each *P. viticola* genome assembly and both *P. halstedii* assemblies are another illustration that the *P. halstedii* 710 genome assembly is significantly less fragmented and more syntenic to *P. viticola* genomes compared with the OS‐Ph8‐99‐BlA4 genome assembly (Figure S2c).

### A network of 354 *in silico* predicted RXLRs led to the selection of 30 ‘core’ conserved RXLR in *P. halstedii*

#### *P. halstedii RXLRs grouped into 40 clusters, including 12 specific to Plasmopara species*

**Figure 1:**
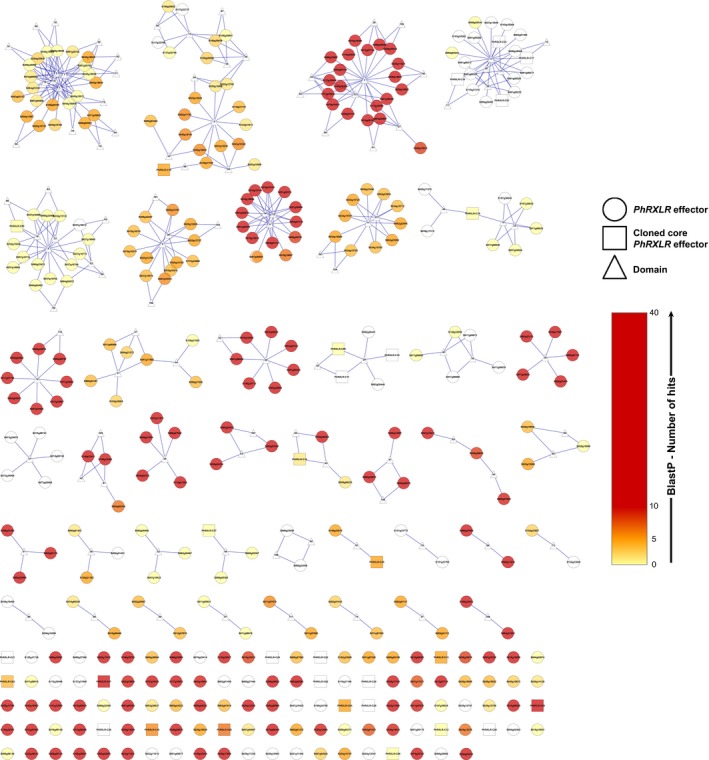
*Plasmopara halstedii* RXLR effector network based on conserved protein domains.The 354 RXLR effector proteins *in silico* predicted are indicated by circles or squares (30 ‘core’ selected RXLRs) and group into 40 connected components (CC) or clusters, that share one or several protein domains indicated by triangles. CC01 to CC40 are sorted by their size and the 112 singletons, meaning proteins that do not share any domain with others, are shown below. The colour code of the RXLR node gives indications of its BlastP score in other oomycete species. The online version (Figure S3, https://ianttoulouseinrafr/EFFECTOORES/webapp/data/clustering/#/) provides additional information on *P. halstedii* RXLRs.

We predicted by SignalP (Nielsen, 2017) 5830 potentially secreted proteins. *In silico* RXLR predictions conducted on this putative secretome led to the selection of 354 RXLR detected by one (298 RXLR), two (17), three (20) or four of the methods used (19) (Figure S1, Table S4). We performed a systematic analysis of protein domains of the 354 RXLR predicted proteins using Mkdom2 (Gouzy *et al*., 1999). The resulting network computed from domain sharing contains 482 edges and 465 nodes, with 354 proteins and 111 protein domains (Figure 1, Figure S3, Table S5). It is composed of 40 connected components (CC: connected parts of the graph in which any node (domain or protein) can be reached from any other node by a path), and 112 singletons that do not share any domain with others (Figures 1, S3). The largest CC01 contains 37 nodes (21 proteins and 16 shared domains) and the smallest only two proteins and one domain (CC29 to CC40).

BlastP analyses of the 354 predicted *P. halstedii* RXLR peptides were performed on all oomycete sequences from the nr protein database (nr 2017/01/10), in order to define their conservation level in other oomycete species (*E*‐value < 10^−4^). BlastP results were most frequent among the Peronosporale oomycetes such as *Phytophthora parasitica* (318), *Plasmopara viticola* (233), and *Ph. infestans* (218), but lowest with the obligate biotroph *Hyaloperonospora arabidopsidis* (51). Here, 124 RXLRs were only found in *P. halstedii* and/or the phylogenetically close *P. viticola* (Sharma *et al*., 2015; Yin *et al*., 2017). Large CC04 (19 proteins) and CC05 (15), and smaller ones (CC09, CC13, CC14, CC16 and six others), contained RXLRs found exclusively in these two Plasmopara species. This suggested the existence of gene families specific to both species and, interestingly, devoid of WY‐domain folds (Boutemy *et al*., 2011), an α‐helical structural motif present in many RXLRs from other Peronosporales (Win *et al*., 2012) (Figure S3, Table S4).

BlastP results give hints on the presumed functions of the proteins grouped in CCs and validate our clustering. For example, the largest CC01 and CC02 consisted each of 21 predicted proteins all homologous to annotated RXLRs of *Ph. infestans*, and that are found in one to six oomycete species (Figures 1, S3). The 20 proteins of CC03 could in fact be *P. halstedii* CRN effectors and not RXLRs. They are very similar to *Ph. infestans* CRN (*E*‐value < 10^−15^) and have the characteristic motifs of CRN effectors LxLFLAK and HVLVVVP (Table S4). They are conserved in at least 12 oomycete species (Figure 1), as reported for oomycete CRN effectors (Schornack *et al*., 2010).

The majority of RXLRs cloned to date have no known functional motifs outside the RXLR region, and have little or no homology in other oomycetes. For these reasons, we estimated that at least 78% (277) of our list (no hit, hit with unknown *Ph. infestans* protein, or hit with *Ph. infestans* RXLR) could be reliable RXLR effectors. Seventy‐one were specific for *P. halstedii* only, and 53 for *P. halstedii* and *P. viticola*.

#### Interpro domains and WY‐domain folds in *P. halstedii RXLR*

To assess our network and to look for known protein domains, Interpro motifs were searched in the 354 *P. halstedii* RXLRs. One‐third (104) of the putative RXLRs matched with Interpro domains, including three with the RXLR phytopathogen effector protein domain IPR031825 (Boutemy *et al*., 2011). The most frequent Interpro domains were the alpha/beta hydrolase fold (IPR029058), the HECT domain (IPR000569) present in E3 ubiquitin‐protein ligases, the DnaJ domain (IPR001623) present in chaperones, the short‐chain dehydrogenase/reductase (IPR002347) and the protein kinase‐like domain (IPR011009), each of these clustered in a similar CC, suggesting that our network using Mkdom2 was relevant. If the proteins having such domains are real effectors, this should hint towards their putative functions during pathogenicity.

An independent search was made in the *P. halstedii* secretome for proteins containing conserved α‐helical WY‐domain folds, a structural domain found in many RXLR effectors from Peronosporales (Win *et al*., 2012), using models described previously (Boutemy *et al*., 2011). Eighty‐six were found, of which 65 were in the 354 RXLR list and contained 1–7 WY‐ domains (mean value per RXLR = 3.7) (Figure S3, Table S4). The 21 WY‐domain fold proteins that were not predicted as RXLR effectors by our workflow did not have a canonical RXLR motif but eight of these displayed an EE(R/E/F) motif, and 11 of these had a BlastP hit with a predicted RXLR of *Ph. infestans*, suggesting that they could be listed as potential effectors.

The 65 *P. halstedii* RXLR having WY‐domain folds were over‐represented in large clusters CC01, CC02, CC06, CC08, and in smaller ones. The proteins of the four large clusters were homologous to annotated *Ph. infestans* RXLRs and had BlastP hits in *Phytophthora* species and in *H. arabidopsidis*, suggesting conserved groups of RXLR with WY‐domain folds among the Peronosporales.

#### Forty‐two P. halstedii RXLR were significantly differentially expressed in infected sunflower

RNA‐seq experiments were performed at two key time points during the *in planta* life cycle of *P. halstedii* pathotype 710: an early, 24 hpi infection stage in roots, both in resistant (TSRM) and susceptible (TS) sunflower genotypes, and then at 10 dpi, during the colonization phase in hypocotyls of the susceptible TS line (Gascuel *et al*., 2016b). Our RNA‐seq data treatment indicated that 325 of the 354 RXLR genes (91.8%) were expressed in at least one of the conditions tested (Figure S3; Tables S6, S7). The RXLR genes expressed in susceptible or resistant roots at 24 h showed few differences (Figure S3), suggesting that plant resistance does not modify their expression at this early stage. Comparison of 10 dpi in hypocotyls with 24 h in susceptible plant roots showed that 42 (indicated by larger node size and node border width, Figure S3) of 325 expressed RXLR were differentially expressed (FDR ≤ 0.05, Table S7). These genes may correspond either to early‐ or late‐induced genes (Figure S3).

#### Polymorphism analysis revealed 58% of conserved RXLR effectors in *P*. *halstedii*

Analysis of RXLR polymorphism among the 17 pathotypes of *P*. *halstedii* sequenced led to the identification of 205 (58%) RXLR proteins showing no amino acid polymorphism, that were defined as *P*. *halstedii* ‘core’ RXLRs. Some medium‐sized CC (CC10, 12–13, 15 and 18) contained mostly core RXLRs, suggesting that they could be organized in gene families. Polymorphism appeared to be randomly spread among the large CC (Figure S3), except for CC01, but RXLRs having WY‐domain folds showed a bias for protein polymorphism (66% polymorphism versus 42% for all RXLRs). This is consistent with the assumption that the molecular stability provided by the core WY‐domain fold, combined with considerable potential for plasticity, is a resource for the pathogen to maintain effectors with virulence activities while evading recognition by the plant immune system (Boutemy *et al*., 2011).

#### Selection of 30 core RXLR effectors conserved in 17 *P*. *halstedii pathotypes*

Thirty core effector proteins representative of the different features encountered in *P. halstedii* RXLRs (i.e. belonging to different CC) and expressed during plant infection were selected for functional studies (Table 1). We selected those having a good chance to be functional effectors (RXLR‐type motif, RXLR Interpro domain, number of prediction methods). They were all expressed in at least two RNA‐seq conditions, and eight were found to be differentially expressed between early and late time points (Figure S3, Table S7). Half of these were in eight CC. Twenty‐four were detected by at least two *in silico* prediction methods; 16 had the RXLR‐(D/E)ER canonical motifs and two others had variant RXLR motifs and WY‐domain folds (Table 1). Half were specific to *P. halstedii*, including four effectors (PhRXLR‐C14, ‐C20, ‐C22 and ‐C29) having no homologs in the published *P. halstedii* sequence, suggesting that our genome assembly is more complete than the previous one, and eight not predicted as being RXLR (Sharma *et al*., 2015) (Tables 1, S4). Six had only homologs in *P. viticola*, and 10 were conserved in at least four oomycete species (Tables 1, S8).

**Table 1:** The 30 selected core RXLR effectors conserved in *Plasmopara halstedii* pathotypes

Core RXLR effector	Gene ID Plhal710r1_	Gene ID in *P.halstedii* proteome^a^	Connected component or singleton	Prediction method	Protein size	Signal Peptide clivage site	RxLR‐like motif: position	dEER‐like motif: position	WY folds	Interpro domain	BlastP hits with oomycete species^b^	qRT‐PCR expression class	qRT‐PCR expression compared to *PhRibS3A*	*P.infestans* INF1 suppression	*in planta* subcellular localization	HR triggering
PhRXLR‐C01	S008g05269	nd	CC04	WI/WH/H/B	138	25	RQLR:42	EER:60	‐	nd	‐	late	lower	+	NC	+
PhRXLR‐C02	S012g07674	CEG35256.1	S	WI/H/B	92	22	RALR:47	‐	‐	nd	‐	spores	stronger	+	NC	‐
PhRXLR‐C03	S016g09123	nd	S	WI/WH/H/B	184	30	RLLR:43	EER:57	‐	nd	4	colonization	lower	+	Nucleus, nucleolus and PM	‐
PhRXLR‐C04	S016g09439	CEG49414.1	S	WI/H/B	119	23	RILR:40	EER:76	‐	IPR031825:RXLR phytopathogen effector protein	5	spores	stronger	+	NC	‐
PhRXLR‐C05	S037g16690	CEG39174.1	CC05	B	244	22	RSLK: 50	EER:67	‐	nd	1	infection	stronger	+	NC	‐
PhRXLR‐C06	S036g16536	CEG45744.1	S	B	168	23	RSAL:46	EER:62	‐	IPR000048:IQ motif, EF‐ hand binding site	1	spores	stronger	+	Golgi bodies (EMS)	‐
PhRXLR‐C07	S064g23400	CEG37861.1	S	WI/H/B	325	20	RGLR:36	EER:73	‐	IPR001237:43kDa postsynaptic protein; IPR011991:Winged helix‐ turn‐helix DNA‐binding domain	13	colonization	stronger	+	NC and nucleolus	‐
PhRXLR‐C08	S068g23969	CEG49461.1	S	B	364	23	‐	EER:58	3	nd	7	spores	lower	+	Golgi and P‐bodies (EMS)	‐
PhRXLR‐C09	S082g26440	CEG36780.1	CC13	WI/WH/H/B	128	23	RLLQ:31RLLR:58	EER:73	‐	nd	1	infection	lower	+	Nucleus and PM	+
PhRXLR‐C10	S082g26452	nd	CC13	WI/WH/H/B	100	20	RSLR:31RLLR:58	EER:72	‐	nd	‐	*colonization* c	stronger	+	Nucleus and nucleolus	+
PhRXLR‐C11	S082g26453	CEG44407.1	CC13	WI/WH/H/B	160	20	KSLQ:31RLLR:58	EER:73	‐	nd	‐	spores	lower	+	Nucleus and PM	+
PhRXLR‐C12	S095g28460	CEG47148.1	S	WI/WH/H/B	173	19	RRLR:41	EER:53	‐	nd	1	late	stronger	+	NC	‐
PhRXLR‐C13	S100g29079	CEG42169.1	S	B	127	26	RVLQ:45	‐	‐	nd	5	infection	lower	‐	Endoplasmic reticulum (EMS)	‐
PhRXLR‐C14	S113g30722	nd	CC20	WI/WH/H/B	89	22	RQLR:30	EER:53	‐	nd	2	spores	stronger	+	NC	‐
PhRXLR‐C15	S118g31182	CEG35764.1	CC02	WI/H/B	564	40	RNLR:44	EER:89	‐	nd	5	colonization	lower	‐	Undefined cytoplasmic bodies	‐
PhRXLR‐C16	S132g32344	CEG43263.1	S	WI/H/B	143	23	RLLR:47	DER:70	‐	IPR031825:RXLR phytopathogen effector protein	6	spores	stronger	+	NC	+
PhRXLR‐C17	S008g05255	CEG49264.1	CC04	WI/WH/H/B	322	28	RQLR:43	EER:60	‐	nd	‐	colonization	lower	+	NC	‐
PhRXLR‐C18	S008g05276	nd	CC04	WI/WH/H/B	250	27	RQLR:45	EER:62	‐	nd	‐	*spores* c	stronger	+	NC	‐
PhRXLR‐C19	S039g17303	CEG47203.1	CC09	WI/H/B	292	23	RLLR:49	EER:62	‐	nd	1	spores	stronger	+	NC	‐
PhRXLR‐C20	S047g19371	nd	S	WI/H/B	107	19	RALR:30	‐	‐	nd	‐	colonization	stronger	‐	Chloroplast	‐
PhRXLR‐C21	S047g19423	CEG45727.1	S	WI/H/B	123	24	RALR:32	EER:63	‐	nd	‐	late	lower	‐	Endoplasmic reticulum (EMS)	‐
PhRXLR‐C22	S060g22597	nd	S	WI/H/B	115	24	RALR:31	‐	‐	nd	‐	spores	stronger	‐	Endoplasmic reticulum (EMS)	‐
PhRXLR‐C23	S096g28489	nd	CC04	WI/WH/H/B	326	28	RQLR:43	EER:60	‐	nd	‐	colonization	lower	+	NC	‐
PhRXLR‐C24	S089g27493	CEG41472.1	S	H/B	132	22	RKLQ:56	EER:72	‐	nd	‐	spores	stronger	‐	Golgi and P‐bodies (EMS)	‐
PhRXLR‐C25	S149g33555	CEG50476.1	CC29	WI/H/B	122	25	RSLR:48	‐	1	nd	5	spores	lower	‐	NC	‐
PhRXLR‐C26	S003g01996	CEG41649.1	S	WI/WH/H	120	30	RNLR:57	EER:75	‐	nd	‐	spores	stronger	‐	Endoplasmic reticulum (EMS)	‐
PhRXLR‐C27	S008g05363	nd	CC27	B	155	25	RRLH:38	‐	‐	nd	1	*colonization* c	stronger	‐	Plastid associated membranes	‐
PhRXLR‐C28	S013g08171	CEG39147.1	S	WI/H	203	25	RHLR:37	‐	‐	nd	11	late	stronger	‐	Cytosolic	‐
PhRXLR‐C29	S023g11993	nd	S	WI/H	143	22	RFLR:50	EER:71	‐	nd	‐	spores	stronger	‐	PM	‐
PhRXLR‐C30	S024g12465	CEG36406.1	S	B	155	27	RRLS:34	EER:62	‐	nd	‐	infection	stronger	‐	PM and tonoplast	‐

a Best reciprocal BlastP hit in the proteome of *P. halstedii* OS‐Ph8‐99‐BlA4 (Sharma et al.,^2015^), threshold E‐value 10^−5^.

b Number of best BlastP hits in oomycete species other than *P. halstedii*, threshold E‐value 10^−4^. No other BlastP hit with an E‐value <10^−4^ were found on nr database of ncbi.

c Pearson correlation below 0,9 (see Fig. 2).

Abbreviations: Connected Component (CC), singleton (S), WI (Win *et al*.,^2012^), WH (Whisson *et al*., 2007), H (Bhattacharjee *et al*., 2006), Blast (B), Nucleocytoplasmic (NC), Plasma membrane (PM), Endomembrane system (EMS).

### The 30 core RXLR effectors group in four expression classes during infection

The expression profiles of the selected effectors were performed by RT‐qPCR in a suspension of *P. halstedii* spores collected from infected cotyledons and in susceptible sunflower seedlings at 3, 7 and 11 dpi with pathotype 710, corresponding, respectively, to early infection, colonization and late infection stages (Figure S4). Nineteen effector genes were more expressed in at least one condition than the *PhRIBS3A* constitutive gene used to normalize the quantity of pathogen (Gascuel *et al*., 2016b) (Table 1). The maximal relative expression values (rev) of these genes were recorded from 1.04 ± 0.33 for PhRXLR‐C07 at 3 dpi to 32.1 ± 4.4 for PhRXLR‐C10, at 7 dpi (Figure S4).

Four expression patterns were observed after data normalization and search for Pearson correlations (Figure 2; Table 1): (i) a group of 14 effector genes showing maximal expression level in spores (blue); (ii) a group of four effectors strongly expressed at early infection stages (orange) linked to the ‘spores’ group; (iii) a ‘colonization’ group of eight effectors peaking at 3 dpi (green); and (iv) a ‘late’ group of four gradually and late‐induced genes (red). The 30 core RXLR effectors displayed different expression patterns during infection, but 26 of these were maximally expressed before 3dpi (Figure 2).

**Figure 2:**
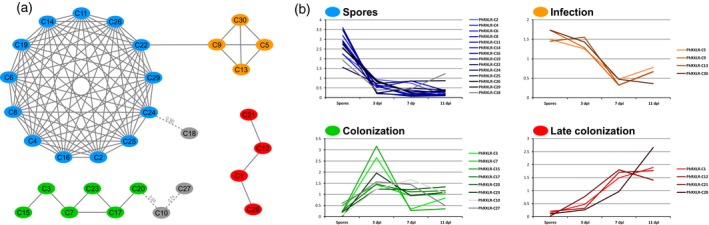
The 30 *Plasmopara halstedii* core RXLR effectors group in four expression classes.Transcript levels of the effectors were measured by RT‐qPCR in *P. halstedii* spores and in infected sunflower seedlings at 3, 7 and 11 dpi corresponding respectively to early infection, colonization and late infection stages, and normalized to *PhRIBS3A* (Gascuel *et al*., 2016b).(a) Co‐expression network based on Pearson correlations (>0.9) revealed four expression groups: (i) a ‘spore’ group in blue showing maximal expression level in spores; (ii) an ‘infection’ group in orange strongly expressed at early infection stages; (iii) a ‘colonization’ group in green peaking at 3 dpi; and (iv) a ‘late colonization’ group in red showing a gradually increase in the level of transcripts during infection. Three RXLR effectors labeled in grey, with Pearson correlations <0.9 were linked to the spore or colonization groups.(b) Expression profiles of core RXLR constituting each expression group are presented. For ease of reading, the relative expression values of each effector were normalized by the mean of the four time points.

### Suppression of pattern‐triggered immunity by *P. halstedii* core RXLR effectors

**Figure 3:**
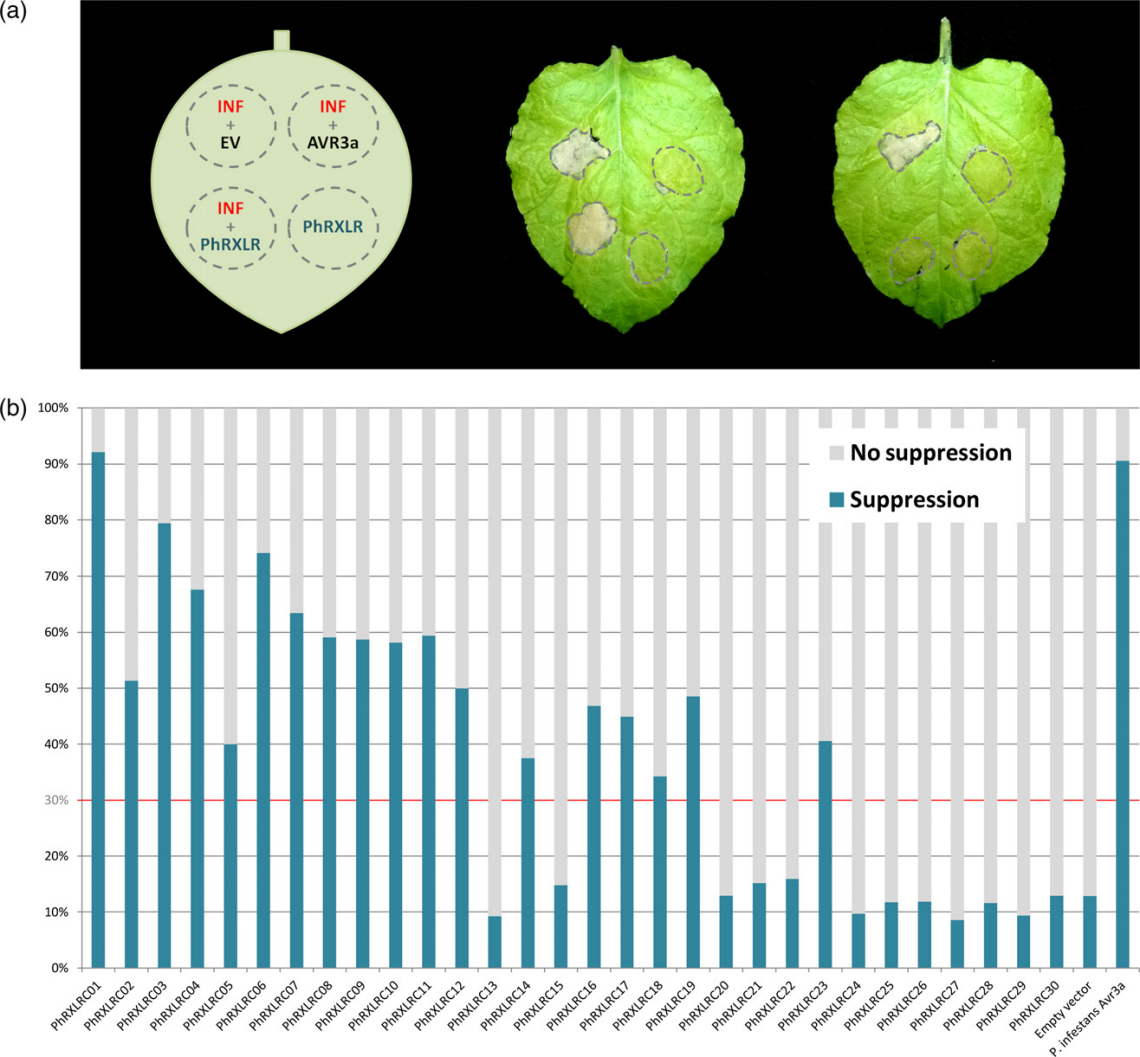
*Plasmopara halstedii* core RXLR effectors suppress pattern‐triggered immunity (PTI) in *Nicotiana benthamiana* leaves.(a) Diagram of PTI suppression assay (left) and results of suppression tests (middle and right leaves). Up‐infiltrated areas are controls performed by co‐infiltrating Infestin1 (INF) from *Phytophthora infestans* with either empty vector (EV) used as negative control (INF1‐triggered cell death) or RXLR effector AVR3a from *Ph. infestans* used as positive control (PTI suppression: no cell death). Middle: example of a construct 35S‐YFP‐PhRXLR core effector that does not suppress PTI, and right, example of a 35S‐YFP‐PhRXLR core effector that does.(b) Histogram showing results of the suppression assays performed with the 30 35S‐YFP‐PhRXLR core effector constructs: Eighteen effectors showed a PTI suppression effect of more than 30%. Symptom development was monitored from 3 to 8 days post‐ infiltration (pictures taken at 7 dpi). The level of suppression was estimated by an average percentage calculated on all infiltrated leaves (*n* > 30).*Ph. infestans* AVR3a reached 90% of suppression in our conditions.

Suppression of pattern‐triggered immunity (PTI) is a good test to estimate the role of a given effector in virulence, and was shown for *Ph. infestans* AVR3a RXLR effector (Bos *et al*., 2006). We tested whether the 30 core RXLR effectors of *P. halstedii* cloned in an expression vector were able to suppress PTI induced by *Ph. infestans* infestin INF1. We first checked that each effector construct induced no cell death per se in *N. benthamiana* leaves. Eighteen *P. halstedii* core RXLR effectors were able to suppress INF1‐induced cell death: five in more than 60% of tested leaves and 13 between 30 and 60% (Figure 3). Therefore, more than half the core effectors tested were able to suppress PTI to some extent*,* suggesting that they could have a role in virulence, validating our choice of effectors. This is noteworthy especially for seven core effectors that showed low expression levels in infected plants (Table 1). Most of those showing INF1 suppression were early‐expressed genes but two of these, PhRXLR‐C01 and ‐C12, displayed late induction patterns (Figure 2), suggesting that PTI suppression is not limited to early induced RXLRs.

### *P. halstedii* core RXLR effectors target the nucleus and other organelles of sunflower cells

**Figure 4:**
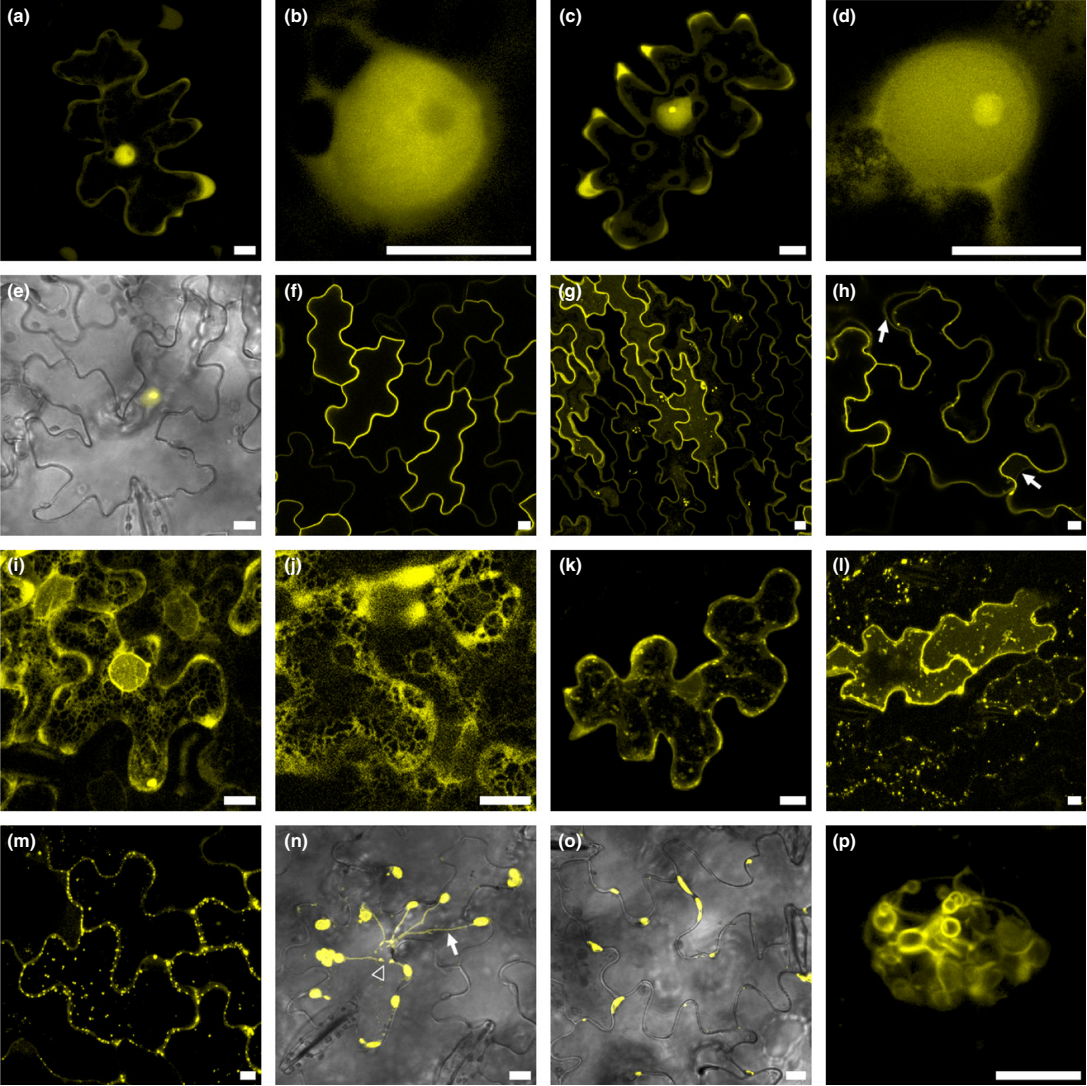
*Plasmopara halstedii* core RXLR effectors target the nucleus and other organelles of sunflower cells.Confocal images of p35S‐YFP‐PhRXLR constructs that were transiently expressed in sunflower leaves by agroinfiltration. Scale bar, 10 μm.(a, b) PhRXLR‐C01 is a nucleocytoplasmic effector not localized in the nucleolus.(c, d) The nucleocytoplasmic effector PhRXLR‐C07 targets nucleolus.(e) PhRXLR‐C10 is exclusively observed in nucleus and nucleolus.(f, g) PhRXLR‐C11 targets plasma membrane and in some cells forms aggregates in nuclei (g, also refer to Figure 5).(h) PhRXLR‐C30 is localized both in plasma membrane and tonoplast (arrows).(i, j) The fluorescent signal of the YFP‐PhRXLR‐C13 construct reveals the endoplasmic reticulum network surrounding the nucleus.(k, l) PhRXLR‐C06 and PhRXLR‐C08 are observed in small cytosolic aggregates corresponding to Golgi bodies (also refer to Figures 5, S6).(m) PhRXLR‐C15 accumulates in undefined and small cytoplasmic bodies observed at the periphery of the cell.(n) PhRXLR‐C20 targets chloroplasts and stromules (arrow) (Caplan *et al*., 2015), tubular membrane extensions of chloroplasts connected to nucleus (arrowhead).(o, p) PhRXLR‐C27 targets aggregates composed of vesicles adjoining chloroplasts also known as plastid‐associated membranes (p is an enlarged view of an aggregate, also refer to Figure 5).

**Figure 5:**
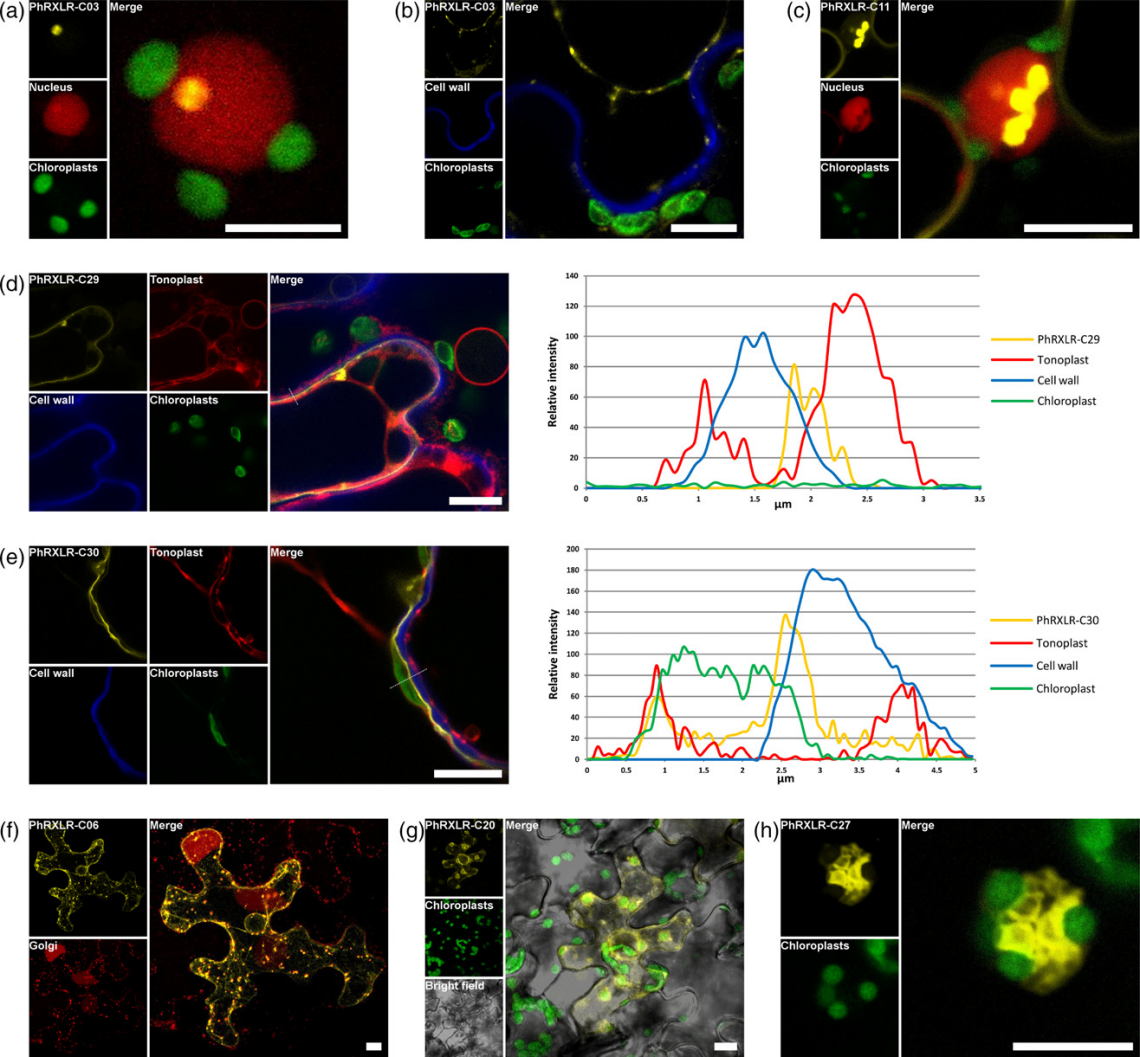
Colocalization studies confirm the original targeting of cell compartments by *Plasmopara halstedii* core RXLR effectors.Confocal images of p35S‐YFP‐PhRXLR and RFP‐tagged marker constructs that were transiently expressed by agroinfiltration in sunflower (a, c, g and h) and *N benthamiana* leaves (b, d−f). Chloroplasts were visualized by autofluorescence and cell walls by calcofluor staining. Scale bar, 10 μm(a) In a nucleus revealed by the nuclear specific marker SV40 large T‐antigen (SVLT) fused to RFP, PhRXLR‐C03 targets exclusively the nucleolus.(b) In a plasmolyzed cell, the YFP‐PhRXLR‐C03 signal is observed in plasma membrane but not in cell wall stained in blue.(c) PhRXLR‐C11 is observed both in plasma membrane and nucleus but, interestingly, no overlapping signal is observed in nucleus between SVLT‐RFP and YFP‐PhRXLR‐C11.(d) YFP‐PhRXLR‐C29 is only observed in plasma membrane, the fluorescence intensity plot computed from the dotted line in the merge panel is shown on the right. This plot shows a single yellow peak of YFP signal reflecting the plasma membrane, located between the cell wall signal in blue and the tonoplast associated signal (aquaporin‐RFP) in red (Saito *et al*., 2002).(e) PhRXLR‐C30 targets both plasma membrane and tonoplast. The fluorescence intensity plot shows two yellow peaks of YFP, the first overlaying the tonoplast signal in red and the second, located between chloroplast and cell wall signals corresponding to the plasma membrane.(f) YFP‐PhRXLR‐C06 signal colocalizes with Golgi bodies revealed by the GntI‐RFP constructs (Essl *et al*., 1999).(g) YFP‐PhRXLR‐C20 signal labels the periphery of the chloroplasts showing the autofluorescent signal in green.(h) YFP‐PhRXLR‐C27 fused protein forms aggregates and labels membranes systemically associated with chloroplasts (in green), also known as plastid‐associated membranes (Andersson *et al*., 2007).

The 30 effectors fused to the C‐terminal end of the yellow fluorescent protein (YFP) were transiently expressed in sunflower and in *N. benthamiana* (Figures 4, 5, S5, S6; Table 1). Thirteen effector−YFP fusions were observed in the nucleus and cytoplasm, as described for YFP alone (Gascuel *et al*., 2016b) (Figure 4a,b). Although the expected sizes of the fusions were checked for 10 of them, we cannot exclude that these localizations were driven by YFP. PhRXLR‐C07 displayed both nucleocytoplasmic and nucleolar localization (Figure 4c,d). Two other effector constructs, PhRXLR‐C03 and PhRXLR‐C10, also targeted nuclei and nucleoli (Figures 4e and 5a). While the signal for PhRXLR‐C10 was only observed in these compartments, PhRXLR‐C03 also displayed some speckles resembling fluorescent signals in the nucleoplasm and a plasma membrane localization (Figure 5b). PhRXLR‐C09 and PhRXLR‐C11 also targeted nuclei and plasma membranes (Figure 4f,g), but the nuclear signals were markedly distinct from those of the effectors described above: an intense YFP signal was observed in undefined large nuclear bodies not targeted by SV40‐red fluorescent protein (RFP) used as a specific nuclear marker (Figures 5c, S6). Respectively, four and three core cloned effectors belonged to CCs CC04 and CC13 (Table 1). It may be noted that all CC04 cloned effectors had the same nucleocytoplasmic localization, whereas three CC13 effectors displayed nuclear localization but only two of these, PhRXLR‐C09 and ‐C11, showed plasma membrane localization (Figures 4f,g, S5, S6, S8).

The non‐nuclear effector constructs targeted diverse plant cell components (Table 1). PhRXLR‐C29 and PhRXLR‐C30 were, respectively, observed in plasma membrane and plasma membrane and tonoplast membrane (Figures 4h, 5d,e). Seven effectors targeted the endomembrane system. The fluorescent signals observed for PhRXLR‐C13, ‐C21, ‐C22 and ‐C26 fusions clearly depicted the endoplasmic reticulum networks (Figures 4i–j, S5). PhRXLR‐C06, ‐C08 and C24 colocalised with a membrane protein that accumulated in Golgi bodies (Figures 4k,l, 5f). PhRXLR‐C08 and ‐C24 also accumulated in aggregates similar to large processing bodies as described up to now only for a fungal effector (Petre *et al*., 2016) (Figure S6). Unprecedented localization patterns for oomycete effectors were also observed for two core effectors. PhRXLR‐C20 localized around chloroplasts and in stromules (Caplan *et al*., 2015), tubular membrane extensions of chloroplasts connected to the nucleus (Figures 4n, 5g, S5). PhRXLR‐C27 targeted aggregates of vesicles systematically linked to chloroplasts, similar to plastid‐associated membranes (Andersson *et al*., 2007) (Figures 4o–p, 5h, S5).

The 30 core effectors were shown to accumulate in sunflower cells and could be tested for recognition by downy mildew‐resistant sunflower lines.

### Five *P. halstedii* core RXLR effectors induce HR responses in sunflower lines resistant to several pathotypes

Transient expression assays of the 30 effector constructs were performed on 16 sunflower lines that have known *Pl R* genes from different clusters, giving resistance from one to 17 pathotypes (Table 2). Four *P. halstedii* effector constructs induced either HR cell death (PhRXLR‐C01, ‐C11 and ‐C16) or a strong discoloration of the infiltrated leaf areas (PhRXLR‐C10) (Figure S7). PhRXLR‐C01 construct induced HR at 4 days post infiltration (dpil) in three differential lines RHA‐274, PMI3 and 803‐1, respectively, resistant to 8, 9 and 16 pathotypes and carrying *Pl2*−*Pl21*,* Pl22* and *Pl5*+ (Table 2). PhRXLR‐C11 and PhRXLR‐C16 constructs both induced cell death in the differential line 803‐1 at 6 dpil. PhRXLR‐C10 construct was recognized in NIL161‐R line, a line carrying *Pl5* on chromosome 13, but not in its susceptible counterpart NIL161‐S. None of the 30 core RXLR constructs induced HR in the seven susceptible lines, or in lines resistant to less than eight pathotypes.

**Table 2:** Functional profiling of 30 core RXLR effectors of *Plasmopara halstedii* for responses in sunflower

Sunflower genotype	Resistance specificity	Genetic locus‐Linkage group	R or S to *P. halstedii* pathotype						R to n pathotypes	Core *P. *halstedii RXLR effectors																														Control
										PhRXLR‐C01	PhRXLR‐C02	PhRXLR‐C03	PhRXLR‐C04	PhRXLR‐C05	PhRXLR‐C06	PhRXLR‐C07	PhRXLR‐C08	PhRXLR‐C09	PhRXLR‐C10	PhRXLR‐C11	PhRXLR‐C12	PhRXLR‐C13	PhRXLR‐C14	PhRXLR‐C15	PhRXLR‐C16	PhRXLR‐C17	PhRXLR‐C18	PhRXLR‐C19	PhRXLR‐C20	PhRXLR‐C21	PhRXLR‐C22	PhRXLR‐C23	PhRXLR‐C24	PhRXLR‐C25	PhRXLR‐C26	PhRXLR‐C27	PhRXLR‐C28	PhRXLR‐C29	PhRXLR‐C30	Empty vector
Inbred lines			100	304	314	334	710	703																																
CAY	no		S	S	S	S	S	S	**0**	**‐**	**‐**	**‐**	**‐**	**‐**	**‐**	**‐**	**‐**	**‐**	**‐**	**‐**	**‐**	**‐**	**‐**	**‐**	**‐**	**‐**	**‐**	**‐**	**‐**	**‐**	**‐**	**‐**	**‐**	**‐**	**‐**	**‐**	**‐**	**‐**	**‐**	**‐**
GB	no		S	S	S	S	S	S	**0**	**‐**	**‐**	**‐**	**‐**	**‐**	**‐**	**‐**	**‐**	**‐**	**‐**	**‐**	**‐**	**‐**	**‐**	**‐**	**‐**	**‐**	**‐**	**‐**	**‐**	**‐**	**‐**	**‐**	**‐**	**‐**	**‐**	**‐**	**‐**	**‐**	**‐**	**‐**
GCX	no		S	S	S	S	S	S	**0**	**‐**	**‐**	**‐**	**‐**	**‐**	**‐**	**‐**	**‐**	**‐**	**‐**	**‐**	**‐**	**‐**	**‐**	**‐**	**‐**	**‐**	**‐**	**‐**	**‐**	**‐**	**‐**	**‐**	**‐**	**‐**	**‐**	**‐**	**‐**	**‐**	**‐**	**‐**
NIL161‐S	no		S	S	S	S	S	S	**0**	**‐**	**‐**	**‐**	**‐**	**‐**	**‐**	**‐**	**‐**	**‐**	**‐**	**‐**	**‐**	**‐**	**‐**	**‐**	**‐**	**‐**	**‐**	**‐**	**‐**	**‐**	**‐**	**‐**	**‐**	**‐**	**‐**	**‐**	**‐**	**‐**	**‐**	**‐**
OEU	no		S	S	S	S	S	S	**0**	**‐**	**‐**	**‐**	**‐**	**‐**	**‐**	**‐**	**‐**	**‐**	**‐**	**‐**	**‐**	**‐**	**‐**	**‐**	**‐**	**‐**	**‐**	**‐**	**‐**	**‐**	**‐**	**‐**	**‐**	**‐**	**‐**	**‐**	**‐**	**‐**	**‐**	**‐**
PSS2	no		S	S	S	S	S	S	**0**	**‐**	**‐**	**‐**	**‐**	**‐**	**‐**	**‐**	**‐**	**‐**	**‐**	**‐**	**‐**	**‐**	**‐**	**‐**	**‐**	**‐**	**‐**	**‐**	**‐**	**‐**	**‐**	**‐**	**‐**	**‐**	**‐**	**‐**	**‐**	**‐**	**‐**	**‐**
TM	no		S	S	S	S	S	S	**0**	**‐**	**‐**	**‐**	**‐**	**‐**	**‐**	**‐**	**‐**	**‐**	**‐**	**‐**	**‐**	**‐**	**‐**	**‐**	**‐**	**‐**	**‐**	**‐**	**‐**	**‐**	**‐**	**‐**	**‐**	**‐**	**‐**	**‐**	**‐**	**‐**	**‐**	**‐**
RHA‐265*	*Pl1*	*Pl1/Pl2*‐LG8	**R**	S	S	S	S	S	**1**	**‐**	**‐**	**‐**	**‐**	**‐**	**‐**	**‐**	**‐**	**‐**	**‐**	**‐**	**‐**	**‐**	**‐**	**‐**	**‐**	**‐**	**‐**	**‐**	**‐**	**‐**	**‐**	**‐**	**‐**	**‐**	**‐**	**‐**	**‐**	**‐**	**‐**	**‐**
CAYRM	*Pl6*	*Pl6/Pl7*‐LG8	**R**	S	S	S	**R**	**R**	**≥3**	**‐**	**‐**	**‐**	**‐**	**‐**	**‐**	**‐**	**‐**	**‐**	**‐**	**‐**	**‐**	**‐**	**‐**	**‐**	**‐**	**‐**	**‐**	**‐**	**‐**	**‐**	**‐**	**‐**	**‐**	**‐**	**‐**	**‐**	**‐**	**‐**	**‐**	**‐**
OEURM	*Pl5*	*Pl5*/*Pl8*‐LG13	**R**	**R**	S	S	**R**	**R**	**≥4**	**‐**	**‐**	**‐**	**‐**	**‐**	**‐**	**‐**	**‐**	**‐**	**‐**	**‐**	**‐**	**‐**	**‐**	**‐**	**‐**	**‐**	**‐**	**‐**	**‐**	**‐**	**‐**	**‐**	**‐**	**‐**	**‐**	**‐**	**‐**	**‐**	**‐**	**‐**
XA*	*Pl4*	nd	**R**	**R**	**R**	S	S	S	**5**	**‐**	**‐**	**‐**	**‐**	**‐**	**‐**	**‐**	**‐**	**‐**	**‐**	**‐**	**‐**	**‐**	**‐**	**‐**	**‐**	**‐**	**‐**	**‐**	**‐**	**‐**	**‐**	**‐**	**‐**	**‐**	**‐**	**‐**	**‐**	**‐**	**‐**	**‐**
Y7Q*	*Pl6‐*	*Pl6*/*Pl7*‐LG8	S	S	S	S	**R**	**R**	**5**	**‐**	**‐**	**‐**	**‐**	**‐**	**‐**	**‐**	**‐**	**‐**	**‐**	**‐**	**‐**	**‐**	**‐**	**‐**	**‐**	**‐**	**‐**	**‐**	**‐**	**‐**	**‐**	**‐**	**‐**	**‐**	**‐**	**‐**	**‐**	**‐**	**‐**	**‐**
GCXRM	*Pl17*	*Pl17*‐LG4	**R**	**R**	**R**	**R**	**R**	**R**	**≥6**	**‐**	**‐**	**‐**	**‐**	**‐**	**‐**	**‐**	**‐**	**‐**	**‐**	**‐**	**‐**	**‐**	**‐**	**‐**	**‐**	**‐**	**‐**	**‐**	**‐**	**‐**	**‐**	**‐**	**‐**	**‐**	**‐**	**‐**	**‐**	**‐**	**‐**	**‐**
TMRM	*Pl17*	*Pl17*‐LG4	**R**	**R**	**R**	**R**	**R**	**R**	**≥6**	**‐**	**‐**	**‐**	**‐**	**‐**	**‐**	**‐**	**‐**	**‐**	**‐**	**‐**	**‐**	**‐**	**‐**	**‐**	**‐**	**‐**	**‐**	**‐**	**‐**	**‐**	**‐**	**‐**	**‐**	**‐**	**‐**	**‐**	**‐**	**‐**	**‐**	**‐**
Ha‐335*	*Pl6*	*Pl6/Pl7*‐LG8	**R**	S	S	S	**R**	**R**	**7**	**‐**	**‐**	**‐**	**‐**	**‐**	**‐**	**‐**	**‐**	**‐**	**‐**	**‐**	**‐**	**‐**	**‐**	**‐**	**‐**	**‐**	**‐**	**‐**	**‐**	**‐**	**‐**	**‐**	**‐**	**‐**	**‐**	**‐**	**‐**	**‐**	**‐**	**‐**
RHA‐274*	*Pl2, Pl21*	Pl1/Pl2‐LG8, Pl21‐LG13	**R**	**R**	**R**	**R**	S	S	**8**	**+**	**‐**	**‐**	**‐**	**‐**	**‐**	**‐**	**‐**	**‐**	**‐**	**‐**	**‐**	**‐**	**‐**	**‐**	**‐**	**‐**	**‐**	**‐**	**‐**	**‐**	**‐**	**‐**	**‐**	**‐**	**‐**	**‐**	**‐**	**‐**	**‐**	**‐**
PMI3*	*Pl22 *=* Pl*_*PMI3*_	nd	**R**	**R**	S	S	S	**R**	**9**	**+**	**‐**	**‐**	**‐**	**‐**	**‐**	**‐**	**‐**	**‐**	**‐**	**‐**	**‐**	**‐**	**‐**	**‐**	**‐**	**‐**	**‐**	**‐**	**‐**	**‐**	**‐**	**‐**	**‐**	**‐**	**‐**	**‐**	**‐**	**‐**	**‐**	**‐**
PSS2RM*	*Pl6, Pl21*	*Pl6/Pl7*‐LG8, *Pl21‐LG13*	**R**	**R**	**R**	S	**R**	**R**	**10**	**‐**	**‐**	**‐**	**‐**	**‐**	**‐**	**‐**	**‐**	**‐**	**‐**	**‐**	**‐**	**‐**	**‐**	**‐**	**‐**	**‐**	**‐**	**‐**	**‐**	**‐**	**‐**	**‐**	**‐**	**‐**	**‐**	**‐**	**‐**	**‐**	**‐**	**‐**
QHP1*	*Pl1, Pl13*	*Pl1/Pl2*‐LG8, *Pl13/16*‐LG1	**R**	**R**	**R**	**R**	**R**	S	**13**	**‐**	**‐**	**‐**	**‐**	**‐**	**‐**	**‐**	**‐**	**‐**	**‐**	**‐**	**‐**	**‐**	**‐**	**‐**	**‐**	**‐**	**‐**	**‐**	**‐**	**‐**	**‐**	**‐**	**‐**	**‐**	**‐**	**‐**	**‐**	**‐**	**‐**	**‐**
PM17*	*Pl5*‐	*Pl5/Pl8*‐LG13	**R**	**R**	**R**	S	**R**	**R**	**13**	**‐**	**‐**	**‐**	**‐**	**‐**	**‐**	**‐**	**‐**	**‐**	**‐**	**‐**	**‐**	**‐**	**‐**	**‐**	**‐**	**‐**	**‐**	**‐**	**‐**	**‐**	**‐**	**‐**	**‐**	**‐**	**‐**	**‐**	**‐**	**‐**	**‐**	**‐**
NIL161‐R	*Pl5*	*Pl5/Pl8*‐LG13	**R**	**R**	**R**	S	**R**	**R**	**14**	**‐**	**‐**	**‐**	**‐**	**‐**	**‐**	**‐**	**‐**	**‐**	**+** ^**1**^	**‐**	**‐**	**‐**	**‐**	**‐**	**‐**	**‐**	**‐**	**‐**	**‐**	**‐**	**‐**	**‐**	**‐**	**‐**	**‐**	**‐**	**‐**	**‐**	**‐**	**‐**
803‐1*	*Pl5*+	*Pl5/Pl8*‐LG13	**R**	**R**	**R**	**R**	**R**	**R**	**16**	**+**	**‐**	**‐**	**‐**	**‐**	**‐**	**‐**	**‐**	**‐**	**‐**	**+**	**‐**	**‐**	**‐**	**‐**	**+**	**‐**	**‐**	**‐**	**‐**	**‐**	**‐**	**‐**	**‐**	**‐**	**‐**	**‐**	**‐**	**‐**	**‐**	**‐**
RHA‐419*	*Pl*_*Arg*_	*Pl*_*Arg*_‐LG1	**R**	**R**	**R**	**R**	**R**	**R**	**17**	**‐**	**‐**	**‐**	**‐**	**‐**	**‐**	**‐**	**‐**	**‐**	**‐**	**‐**	**‐**	**‐**	**‐**	**‐**	**‐**	**‐**	**‐**	**‐**	**‐**	**‐**	**‐**	**‐**	**‐**	**‐**	**‐**	**‐**	**‐**	**‐**	**‐**	**‐**
broad‐spectrum resist germplasm																																								
HAS6	nd	nd	**R**	**R**	**R**	**R**	**R**	**R**	**17**	**+**	**‐**	**‐**	**‐**	**‐**	**‐**	**‐**	**‐**	**‐**	**‐**	**+**	**‐**	**‐**	**‐**	**‐**	**‐**	**‐**	**‐**	**‐**	**‐**	**‐**	**‐**	**‐**	**‐**	**‐**	**‐**	**‐**	**‐**	**‐**	**‐**	**‐**
HAS85	nd	nd	**R**	**R**	**R**	**R**	**R**	**R**	**17**	**+**	**‐**	**‐**	**‐**	**‐**	**‐**	**‐**	**‐**	**+**	**‐**	**+**	**‐**	**‐**	**‐**	**‐**	**‐**	**‐**	**‐**	**‐**	**‐**	**‐**	**‐**	**‐**	**‐**	**‐**	**‐**	**‐**	**‐**	**‐**	**‐**	

Transient expression assays of the 30 effector constructs 35S‐YFP‐PhRXLR were performed on seven sunflower lines susceptible to all *P halstedii* pathotypes, 16 lines which have known *Pl* genes from different clusters, giving resistance from one to 17 pathotypes, and 2 lines, HAS6 and HAS85, carrying as yet unpublished resistances towards all 17 pathotypes. Sunflower lines* are differential lines used for phenotyping *P halstedii* pathotypes (Gascuel *et al*., 2015) and whose resistance (R) or susceptibility (S) is known towards the 17 pathotypes. The R or S phenotype of the other lines was determined with 6 *P halstedii* pathotypes. Following agroinfiltration, the infiltrated leaf sectors were noted at 4, 7 and 11 days using a scoring necrotic scale (Gascuel *et al*., 2016b). The experiments were performed at minimum twice, and three times for positive responses, on 2 leaves of 12 plants of each sunflower line with the effector and with the control. Mean scores were calculated and scores of 4 to 5 corresponded to HR responses (+), and of 3 to strong discoloration HR‐ like reaction (+^1^).

To check whether new broad‐spectrum resistances could be revealed by core RXLR effectors, two lines, HAS6 and HAS85, carrying as yet unpublished resistances were tested with the 30 effector constructs (Table 2). PhRXLR‐C01 and PhRXLR‐C11 constructs induced HR in both, but HAS85 revealed another HR‐inducing effector, PhRXLR‐C09. The germplasm lines HAS6 and HAS85, resistant to all 17 pathotypes, showed HR in response to several core effectors.

### HR induced by PhRXLR‐C01 and the *Pl22* resistance gene co‐segregate in sunflower

**Figure 6:**
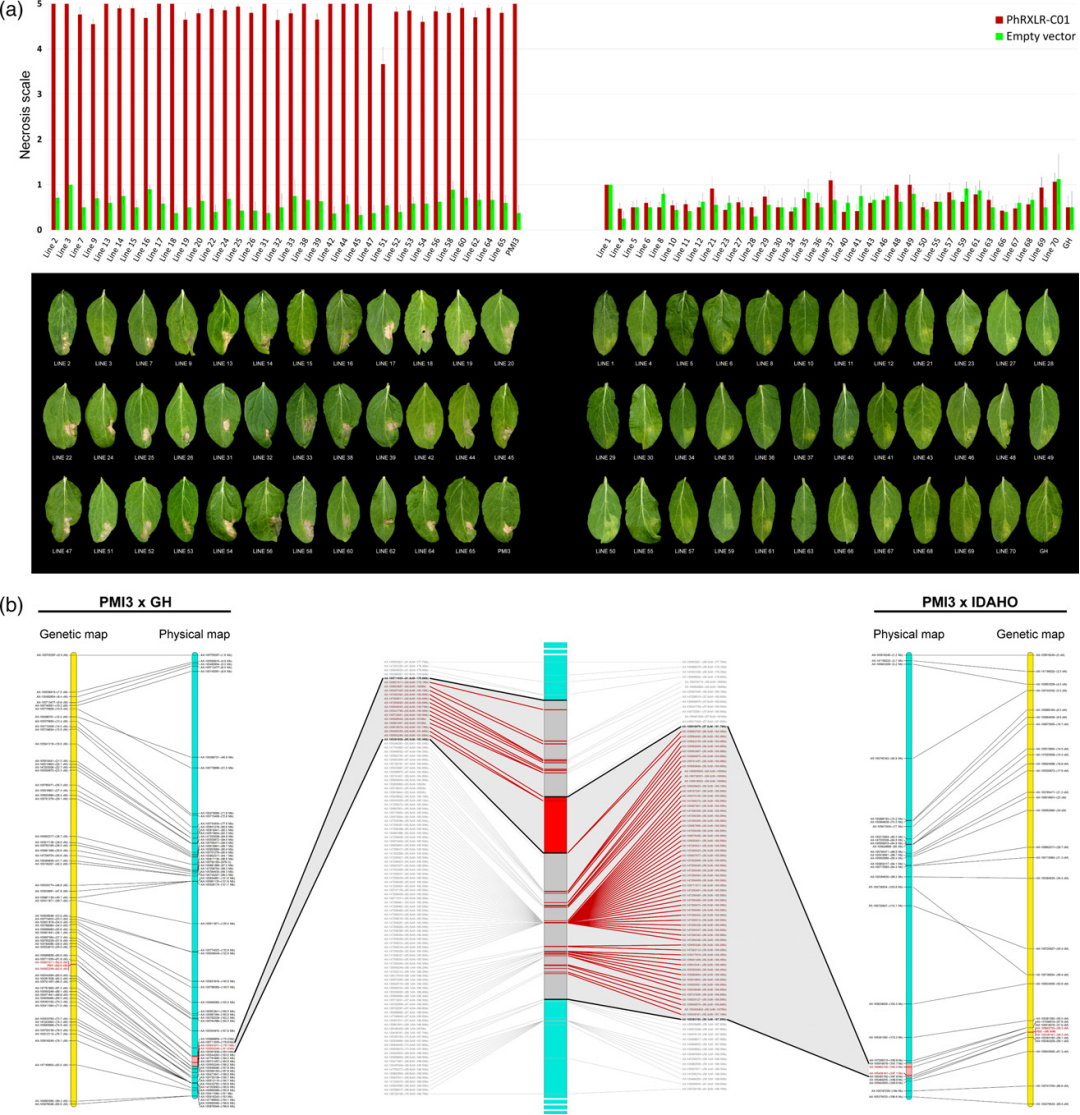
The *Pl22* downy mildew resistance gene and recognition of the core effector PhRXLR‐C01 co‐segregate on chromosome 13 of sunflower.(a) Thirty‐five *Pl22* resistant (left graph) and 35 susceptible (right graph) F3 progenies of the cross PMI3 (*Pl22*) × GH (susceptible) were tested by agroinfiltration with the core RXLR effector PhRXLR‐C01 and with the empty vector as control. The infiltrated leaf sectors were scored at 4, 8, and 12 days post infiltration using a scoring necrotic scale from 0 (no visible effect) to 5 (confluent HR) (Gascuel *et al*., 2016b). Error bars represent means ± SD (two independent biological replicates, each performed on two leaves of 12 plants). Pictures of corresponding infiltrated leaves with PhRXLR‐C01 are shown below each graph.(b) Genetic (yellow) and physical (blue) maps of the *Pl22* resistance gene on chromosome 13, obtained with PMI3 × GH (left map) and with PMI3 × Idaho (right map) crosses, only the non‐redundant SNP markers are indicated. The central panel is an enlarged view of the physical map showing all the genetically redundant but physically distant markers for both crosses. The chromosomal region in red containing *Pl22* is defined by the overlap between both maps. The polymorphic SNP markers of the sunflower AXIOM array denominated AX‐xxxxxxxxx are indicated with their genetic and physical positions on the sunflower XRQ genomic sequence (Badouin *et al*., 2017).

PhRXLR‐C01 construct triggers an HR in the line PMI3, carrying a so‐far‐unmapped resistance gene, *Pl22*. We tested with PhRXLR‐C01 construct the F3 progenies of a cross involving the line PMI3 as the resistant parent (PMI3 × GH), segregating for the single dominant gene *Pl22*. HR cell death in response to PhRXLR‐C01 infiltration was shown by all the homozygous resistant progenies carrying *Pl22* but none of the homozygous susceptible (Figure 6a). The (PMI3 × GH) progenies therefore indicate an absolute link between the presence of the *Pl22* resistance gene in a plant and its reaction to the effector construct PhRXLR‐C01. This trait was genotyped on an AXIOM sunflower genotyping array of 49 449 SNPs (Mangin *et al*., 2017) in a region of 2.7 Mb of chromosome 13 (Figure 6b). Another F3 population segregating for *Pl22* (PMI3 × Idaho) also segregated for *Pl17*, present in Idaho on LG4 (Qi *et al*., 2015), when tested with pathotype 710, to which PMI3 is susceptible. As Idaho did not respond to PhRXLR‐C01, when the F3 (PMI3 × Idaho) population was scored with PhRXLR‐C01 construct, we considered that the effector reaction indicated the presence or absence of *Pl22*. Increasing the size of the mapping population, and the number of polymorphic markers coming from different sunflower lines made it possible to map the PhRXLR‐C01 recognition and *Pl22* resistance to an overlapping region of 1.7 Mb on chromosome 13, between markers AX‐105918079 at 181.7 Mb and AX‐105391938 at 183.4 Mb from the corresponding XRQ genomic region (Badouin *et al*., 2017) (Figure 6b). This region covers 39 genes expressed during *P. halstedii* infection including two putative HR‐like lesion‐inducers and eight NBS‐LRR putative resistance genes that are candidates for the *Pl22* gene (Table S9).

## Discussion

In this study, we confirmed that effectoromics on core downy mildew effectors can speed up characterization of broad‐spectrum disease resistance genes in sunflower (Dangl *et al*., 2013). We started our analyses from a comprehensive network analysis of *P. halstedii* RXLR effectors. We searched for connected groups sharing uncharacterized domains, especially when no clue is given by the sequence itself, as is generally the case for oomycete RXLRs, and true for 250 *P. halstedii* RXLR having no Interpro domain. This approach led us to find groups of connected effectors sharing still unknown functional domains. This should also help to understand evolution of an effector family towards acquisition of new allelic variants that may overcome plant resistance. For example, the five effector genes of CC13 were physically close on the same genomic scaffold and probably resulted from duplication events (Gascuel *et al*., 2016a), and were combinations of two protein domains (Figure S8). Their subcellular localizations were different: PhRXLR‐C10 showed nuclear and nucleolar localization similar to the polymorphic effector of the same CC (s082 g26441) previously known as PhRXLR02 (Gascuel *et al*., 2016b), while both PhRXLR‐C09 and ‐C11 were targeted to the plasma membrane and nucleus (but not to the nucleolus). The fact that these related effectors are recognized by different resistances in sunflower and are localized differently might reflect subfunctionalization events in order to create variant forms of effectors, escaping plant recognition.

A large group of 205 core RXLR effectors was revealed in *P. halstedii*. This is probably due to the recent evolution of pathotypes that appeared over only 40 years and to a predominant reproduction by homothallic selfing, causing reduced genetic variation and increased linkage disequilibrium compared with heterothallic oomycete species (Ahmed *et al*., 2012; Gascuel *et al*., 2015). In a previous study on seven *P. halstedii* pathotypes, we estimated the percentage of polymorphic genes encoding 54 RXLR and CRN effectors twice as high as for randomly selected noneffector genes (Gascuel *et al*., 2016a), suggesting a bias towards polymorphism in effector genes.

RXLRs containing the conserved α‐helical WY‐domain fold, are restricted to Peronosporales (Win *et al*., 2012), *Phytophthora* species possessed up to 44%, and *Hyaloperonospora* 34%. In *P. halstedii* we estimated these at 18–23%, if we include those not predicted by the RXLR workflow. It should be noted that none of the five HR‐inducing *P. halstedii* RXLR effectors displayed WY‐domain folds.

In oomycetes, the notion of core effectors is recent and not precisely documented, probably because of the lack of pathotype sequences (Cooke *et al*., 2012), in contrast with plant bacterial pathogens. For example, *Ralstonia solanacearum* strains, the agent of plant bacterial wilt, typically possess 60–75 effectors including 32 core effectors present in 10 strains, presumably present in the *R. solanacearum* ancestor before phylotype divergence (Deslandes and Genin, 2014), this could also be the case for *P. halstedii* core effectors. For pathogens with many invariant effectors, an additional feature to define a ‘core effector’ could be the expression in all pathotypes.

An original set of 30 RXLR core effectors was studied functionally, including 10 effectors absent of the former *P. halstedii* proteome (Sharma *et al*., 2015), but for six of which we provide functional data. Eighteen effectors in total showed a PTI suppression effect, but we have to be cautious because of possible overestimated rates. Effectors triggering HR have suppression rates higher than 45%, PhRXLR‐C01 being over 90%, suggesting that they also play a role in pathogen virulence. The 18 INF1‐suppressors showed either a nuclear localization in sunflower (16 effectors, including the five HR‐triggers) or in Golgi bodies (two effectors); conversely the effectors localized in plasma membrane, endoplasmic reticulum, or chloroplasts did not suppress INF1‐induced PTI, suggesting that they could have another function in pathogenicity, impairing plant cell metabolism and secretion via the endomembrane system and increasing pathogen fitness.

The unprecedented localization of two RXLR effectors in close structures connected to chloroplasts reinforces the central role of plastids in early plant defense (Caplan *et al*., 2015; Zabala *et al*., 2015). *Arabidopsis thaliana* chloroplasts were targeted with bacterial effectors of *Pseudomonas syringae* (Zabala *et al*., 2015). Chloroplast stromules were shown to be induced in effector‐triggered immunity (ETI) in *A. thaliana* and *N. benthamiana* in response to bacterial and viral effectors, but not during PTI (Caplan *et al*., 2015). The two chloroplastic *P. halstedii* RXLRs were early induced and did not suppress PTI. Similar to the effect of bacterial effectors, they might prevent a chloroplastic reactive oxygen burst, blocking plant immunity (Zabala *et al*., 2015).

Although all *P. halstedii* pathotypes possess the set of core effector genes, some of these still trigger susceptibility in plants having a matching *R* gene. Plant susceptibility could be explained by either lack of expression of the effector, or by suppression of its function by another effector present in these pathotypes. RXLRs of the oomycete *Ph. sojae* were shown to interact functionally: early‐expressed effectors suppress the cell death triggered by later ones (Wang *et al*., 2011). As PhRXLR‐C01 is a late effector in *P. halstedii*, it is possible that pathotypes triggering plant susceptibility express RXLRs able to suppress the action of PhRXLR‐C01, thereby preventing resistance.

PhRXLR‐C01 effector triggered HR in sunflower lines carrying different *R* genes. In the guard model (Jones and Dangl, 2006) the recognition of the effector (considered as avirulence protein) often occurs through the targeting of a guardee protein, which is guarded by the so‐called R guard protein responsible for resistance activation. A guardee protein targeted by PhRXLR‐C01 could be present in different sunflower lines, but resistance would be activated only in the presence of matching R guards encoded by the *Pl* genes. For Pl22 resistance in PMI3, a single R protein might be involved, comparable with *Arabidopsis thaliana* RPP1 R protein that cumulates two functions: the recognition of the oomycete *H. arabidopsidis* ATR1 effector protein and the activation of resistance signaling (Rehmany *et al*., 2005; Jones *et al*., 2016).

We showed that HR triggered by PhRXLRC01 core effector co‐segregated with resistance controlled by the *Pl22* gene. This is not always the case as reported for *Bremia lactucae* RXLR effectors tested in the nonhost lettuce *Lactuca saligna* (Giesbers *et al*., 2017). To our knowledge, no other avirulence/resistance gene couple such as *PhRXLRC01*/*Pl22* has been identified in sunflower before.

The core effector recognition screening turns out to be a successful strategy to accelerate and diversify resistance gene identification in plant germplasm in a complex crop genome such as sunflower. Screening larger populations with the HR‐inducing effectors should ultimately define genetically a few candidate *R* genes, that could be functionally characterized in a transient assay involving the effector. Transient assays of cloned effectors in plants are much easier to perform than long inoculation tests with a living obligate biotrophic oomycete in quarantine conditions. Broad‐spectrum resistance, the molecular basis of which is still largely unknown, is likely to be more sustainable than resistance effective against only a few pathotypes that has been overcome in the past. The identification of such resistances is an important challenge in all crops (Dangl *et al*., 2013) to decrease the use of pesticides. In addition, compared with resistance gene pyramiding used until now, which may have some negative effects, it may be a more sustainable and economically acceptable strategy to maintain crop yield.

## Experimental Procedures

### *P. halstedii* pathotypes and sunflower lines

Sporangia and spores of the 17 reference *P. halstedii* pathotypes (100, 300, 304–10, 304–30, 307, 314, 330, 334, 700, 703, 704, 707, 710, 714, 717, 730, 774) were collected from the susceptible sunflower variety Peredovik and their pathotypes checked on differential sunflower lines (Gascuel *et al*., 2015). All pathotypes except 330 were collected in France (of which 11 are present in North America), and pathotype 330 absent from France was collected from North America. All sunflower lines used are described in Table 2 and Table S10 and grown as described in Gascuel *et al*. (2016b).

### Genome assembly of *P. halstedii* pathotype 710

Paired‐end and mate‐pair libraries (Table S1) made from pathotype 710 spore DNA were sequenced at the GeT‐PlaGe facility (Toulouse, France) and by Eurofins MWG Operon (Ebersberg, Germany). Paired‐end reads were cleaned and transformed into virtual long reads using boost‐r (http://lipm-bioinfo.toulouse.inra.fr/download/boost-r), and then assembled into contigs using Celera assembler. After removal of contigs included in longer contigs, scaffolding of contigs using mate‐pair read information was carried out with LYNX scaffolder (http://lipm-bioinfo.toulouse.inra.fr/download/lynx). The final assembly contains 745 scaffolds spanning 67.6 Mb (N50 of 496 278 bp and 3% of ‘N’). Genome annotation was performed with EuGene (Figure S1).

### Assessment of the taxonomic origin of sequence data and assembly contigs

Raw Illumina reads were first analysed on the NCBI Sequence Read Archive (SRA) web site (Table S11). Here, 2.3% of the paired‐end reads (SRR7441834) appeared to be related to *Gallus gallus* reads and 0.79% to *Helianthus annuus*. GC percent, taxonomy relationships, Illumina reads coverage, intersection with RNA‐seq polyA data, and similarity with *Plasmopara* public data were computed for the 802 scaffolds of the raw assembly (Table S12). An integrated expert analysis of the results allowed the classification of 182 short contigs as the probable contaminant contigs (minimum length: 1003; maximum length: 4106 nt; total nucleotides: 277 055). Seven contigs were classified as ‘dubious’ for 34 260 nucleotides. The remaining 613 contigs (67 404 527 nucleotides in total) present features expected for *Plasmopara halstedii*. During the genome submission to NCBI, 57 scaffolds were detected as contaminations (86 067 nucleotides) and were removed in the public version of the genome (745 scaffolds). Only 48 scaffolds were detected as contamination by both protocols, illustrating the difficulty of the analysis.

### Identification of *P. halstedii* RXLR effectors and WY‐fold containing proteins in *P. halstedii* genome 710 and their corresponding genomic sequences in 17 pathotypes

The secretome of the *P. halstedii* pathotype 710 was defined from the 36 972 predicted proteins in the genome described above. Signal peptides (SP) were predicted using three versions of SignalP (V2.0, 3.0 and 4.1) using default settings (Nielsen, 2017). RXLR predictions were performed using BlastP, HMM and heuristic approaches (Bhattacharjee *et al*., 2006; Whisson *et al*., 2007; Win *et al*., 2012) with a workflow for RXLR detection developed on the Galaxy web platform (Cock and Pritchard, 2014). BlastP was made against an RXLR reference dataset and only hits with an *E*‐value <10^−5^ were retained. This reference dataset consisted of 2663 proteins described as (putative) RXLR in 14 oomycetes and fungi species (Table S13). Proteins containing WY folds were searched in the whole proteome using HMMER3 and an HMM model previously described (Boutemy *et al*., 2011). In total, 131 proteins containing 1–28 WY domains were found, among these, 86 had a SP prediction and 65 were present in the 354 RXLR list including two conserved cloned RXLRs. The 16 other pathotypes of *P. halstedii* were sequenced by Illumina technology (100 X), reads were mapped on the 710 genome assembly and RXLR polymorphism determined (Figures S1 and S3, Table S4).

### Protein clustering of *P. halstedii* putative RXLRs based on shared Mkdom2 domains

We performed a systematic analysis of protein domains on the 354 predicted RXLR proteins from pathotype 710 on their processed form, meaning that it is devoid of their signal peptide (Table S4), using Mkdom2 (Gouzy *et al*., 1999). A network was built from the Mkdom domains shared by proteins in the form of a bipartite graph where there are two types of nodes: domains and proteins. There is an edge between a domain and a protein if the domain has been identified in the protein. Domains belonging to only one protein were discarded.

Graphical representation of the network and mapping of metadata on the nodes was performed with Cytoscape 3.5 (Shannon *et al*., 2003). The web version of the network was first created with the web exporter of Cytoscape (Ono *et al*., 2014) and tuned for our purpose. It uses the JavaScript library Cytoscape.js (Franz *et al*., 2016) for rendering the network. Interpro domains were identified with InterproScan 5 (Jones *et al*., 2014). Web graphical representation of the Mkdom and Interpro domains has been performed thanks to the neXtProt feature viewer Biojs package (Gomez *et al*., 2013).

### RNA‐seq experiments

RNA extractions were performed as previously described (Gascuel *et al*., 2016b). RNA‐seq libraries were prepared at the GeT‐PlaGe facility (Toulouse, France) from mRNA selected using poly‐T beads, according to the Illumina TruSeq Stranded mRNA sample prep kit protocols. RNA‐seq read pairs were mapped on *P. halstedii* 710 genome using the glint software with parameters set as follows: matches ≥50 nucleotides, ≤4 mismatches, no gap allowed, only best‐scoring hits taken into account (Table S6). Ambiguous matches (same best score) were removed. Statistical treatment of the mapped reads was carried out with under [R] environment using the DESeq package (Love *et al*., 2014). Independent filtering and outlier detection methods proposed by the package were used. Genes presenting a false‐discovery rate (FDR)‐corrected *P*‐value ≤0.05 were considered as differentially expressed between two given conditions. The overwhelming number of sunflower reads in the samples collected at the beginning of infection by *P. halstedii* (24 hpi) compared with the *P. halstedii* ones implies to consider results on differential expression with caution (Table S6).

### RT‐qPCR experiments and cloning of the 30 conserved RXLR effectors

RT‐qPCR experiments were performed from three biological replicates as described previously (Gascuel *et al*., 2016b). Pearson correlations between expression patterns were calculated with a threshold of 0.9. cDNA from *P. halstedii* pathotype 710 spores was used as a matrix to amplify the core effectors, with Phusion High‐Fidelity *Taq* DNA polymerase. Primers were designed with Primer3 software on the coding sequence of each gene with its predicted signal peptide deleted (Table S14). PCR products were cloned in a pBin19‐35S‐YFP‐GW Gateway expression vector (denominated as empty vector) to create fusion proteins under the control of the 35S promoter with YFP in N‐terminal part of the protein (Gascuel *et al*., 2016b).

### *Agrobacterium*‐mediated transient expression experiments, subcellular localization of effector candidates and infestin cell death suppression assays

Transient expression of YFP‐PhRXLR constructs were performed as previously described (Gascuel *et al*., 2016b), with an OD_600_ of 1. Similar procedures were used for suppression assays in *N. benthamiana*, except, for expression of AVR3a (construct pGR106‐AVR3a^KI^; Bos *et al*., 2006) or YFP, the final OD_600_ of recombinant *A. tumefaciens* strains was 1 in induction buffer. Infiltration sites were challenged 1 day after infiltration with recombinant *A. tumefaciens* carrying p35‐INF1 (construct pCB302‐1 INF1; Bos *et al*., 2006) at a final OD_600_ of 1 in induction buffer. All suspensions were incubated for 4 h prior to infiltration. Symptom development was monitored from 3 to 8 days after infiltration (pictures taken at 5 dai).The level of suppression was estimated by an average percentage calculated on all infiltrated leaves (>30).

### Confocal analyses

Confocal analyses were performed as described (Gascuel *et al*., 2016b) either in *N. benthamiana*, or in sunflower lines CAY and Peredovik. For colocalization studies, *N*‐acetyl glucosaminyltransferase I (GntI) a Golgi‐resident type–II membrane protein that accumulates in Golgi bodies was used (Essl *et al*., 1999). SV40‐RFP was used as a specific nuclear marker (Goldfarb *et al*., 1986), and *A. thaliana* aquaporin named gamma‐TIP was used as a tonoplast marker (Saito *et al*., 2002). Calcofluor was used to stain cell walls, and chloroplast autofluorescence was visualized upon YFP excitation.

### HR phenotype scoring

Phenotypes of infiltrated sectors were scored at 4, 8 and 12 days post agroinfiltration on a minimum of 12 plants (24 leaf sectors) per genotype, repeated twice, using a cell death index scale (Gascuel *et al*., 2016b).

### Population segregation tests and genetic mapping protocol

Downy mildew tests performed on F3 progenies (PMI3 × GH) and (PMI3 × Idaho) were performed with races 703 and 710, respectively, and indicated segregation for single dominant genes in PMI3 against pathotype 703 (*Pl22*) and in Idaho against pathotype 710 (*Pl17*): PMI3 × GH: 58 homozygous resistant: 121 segregation: 66 homozygous susceptible (*X*²: 0.559 nsec), PMI3 × Idaho: 51 homozygous resistant : 81 segregation: 45 homozygous susceptible (*X*²: 1.395 nsec). The gene in Idaho is no doubt *Pl17* mapped on chromosome 4 (Qi *et al*., 2015), as the line used, HA458, is derived from the same origin as Idaho and both resist to all virulent pathotypes identified in France and in Northern America. In this cross, segregation for the gene in PMI3 could not be studied as Idaho is also resistant to pathotype 703. Tests with PhRXLR‐C01 were performed on 48 homozygous‐resistant and 49 homozygous‐susceptible F3 progenies of the PMI3 × GH and 70 (12 homozygous HR+, 27 homozygous HR− and 31 segregating HR+/HR−) of PMI3 × Idaho (12 plants tested by F3). For genotyping, each F3 family was grown in a field nursery (eight plants/family), and DNA was extracted from eight leaf discs, one per plant. DNA extractions and genotyping experiments on AXIOM sunflower genotyping array were performed and analysed as described (Mangin *et al*., 2017). Genetic mapping was performed with CarthaGene (de Givry *et al*., 2005) using the following parameters: Haldane metrics, LOD score threshold of 3, and genetic distance of 0.3 (Tables S15, S16).

## Authors’ contributions

YP, LB, and CP did most experiments, with contributions from FV and LG. YP, LB and LG analysed the data and YP did most figures with contributions of LB. LL and JG performed the genome assembly and annotations, and the mapping of RNA‐seq reads. MM and LC with advice of JG performed the RXLR network. OB was involved in the Illumina sequencing of the *P. halstedii* genomes and of RNA‐seq reads. DR analysed the RNA‐seq data. FV produced and phenotyped sunflower lines and F3 progenies. FV, YP and JG contributed to the manuscript. LG conceived the project and the research plans and wrote the manuscript. All authors read and approved the final manuscript.

## Conflict of Interest

The authors declare no conflicts of interest.

## Supporting information


**Figure S1.** *Plasmopara halstedii* RXLR selection workflow and RXLR number detected by the different methods used.

**Figure S2.** Comparisons of *P. halstedii* genome assemblies with *Plasmopara viticola* genome assemblies.

**Figure S3.** Online version of *P. halstedii* RXLR effector network* or pdf screenshots of the network images.*https://ianttoulouseinrafr/EFFECTOORES/webapp/data/clustering/#/

**Figure S4.** Expression analysis by RT‐qPCR of 30 *P. halstedii* core RXLR effectors in spores and during sunflower infection.

**Figure S5.** Subcellular localizations of the 30 *P. halstedii* core RXLR effectors in sunflower cells.

**Figure S6.** Colocalization studies of YFP‐PhRXLR core effector constructs with RFP‐tagged markers.

**Figure S7.** Recognition of *P. halstedii* RXLR core effectors in resistant sunflower lines.

**Figure S8.** Subfunctionalisation of the *P. halstedii* RXLR family of Connected Component 13.

**Table S1.** Statistics, description and SRA accession numbers of raw genomic data of *P. halstedii* pathotype 710.
**Table S2.** *P. halstedii* 710 genome completeness analyses performed by BUSCO 3.0.2.
**Table S3.** Best reciprocal hit Blast between the predicted proteins from *P. halstedii* 710 and from *P. halstedii* OS‐Ph8‐99‐BlA4 (Sharma *et al*., 2015).
**Table S4.** Description of the 354 *P halstedii* RXLR effectors.
**Table S5.** Mkdom domains and Interpro hits for the 354 *P. halstedii* RXLRs.
**Table S6.** Statistics, description and SRA accession numbers of raw RNA‐seq data.
**Table S7.** RNA‐seq results for the genes of *P halstedii* pathotype 710 upon sunflower infection.
**Table S8.** BlastP best hits in oomycete species of the 30 *P. halstedii* core RXLR effectors.
**Table S9.** Sunflower candidate genes for *Pl22* resistance gene expressed upon infection by *P. halstedii*.
**Table S10.** Sunflower lines used in this study.
**Table S11.** Taxonomy analysis of the *P. halstedii* 710 sequenced reads.
**Table S12.** General features of *P. halstedii* 710 genomic scaffolds.
**Table S13.** List of oomycetes and fungi used to construct the reference dataset of RXLRs for BlastP analyses.
**Table S14.** Primer sequences used for cloning and RT‐qPCR experiments.
**Table S15.** Mapping data file of PMI3xGH F3 progenies used for genotyping Pl22 resistance.
**Table S16.** Mapping data file of PMI3xIDAHO F3 progenies used for genotyping Pl22 resistance.

**  **

## Data Availability

Genome assembly and annotation data of the *P. halstedii* pathotype 710 have been deposited in DDBJ/ENA/GenBank under accession number PDFJ00000000.1 and are available at https://doi.org/10.15454/1.5083166732793875e12. Illumina raw data of genome and RNA‐seq reads have been deposited in the SRA under study accession number SRP151444 (Tables S1, S6).

## References

[tpj14157-bib-0001] Ahmed, S. , de Labrouhe, D.T. and Delmotte, F. (2012) Emerging virulence arising from hybridisation facilitated by multiple introductions of the sunflower downy mildew pathogen *Plasmopara halstedii* . Fungal Genet. Biol. 49, 847–855. 22789868 10.1016/j.fgb.2012.06.012

[tpj14157-bib-0002] Andersson, M.X. , Goksor, M. and Sandelius, A.S. (2007) Membrane contact sites: physical attachment between chloroplasts and endoplasmic reticulum revealed by optical manipulation. Plant Signal. Behav. 2, 185–187. 19704692 10.4161/psb.2.3.3973 PMC2634053

[tpj14157-bib-0003] As‐sadi, F. , Carrere, S. , Gascuel, Q. ***et al.*** (2011) Transcriptomic analysis of the interaction between *Helianthus annuus* and its obligate parasite *Plasmopara halstedii* shows single nucleotide polymorphisms in CRN sequences. BMC Genom. 12, 498. 10.1186/1471-2164-12-498 PMC3204308 21988821

[tpj14157-bib-0004] Bachlava, E. , Radwan, O.E. , Abratti, G. , Tang, S.X. , Gao, W.X. , Heesacker, A.F. , Bazzalo, M.E. , Zambelli, A. , Leon, A.J. and Knapp, S.J. (2011) Downy mildew (Pl (8) and Pl (14)) and rust (R (Adv)) resistance genes reside in close proximity to tandemly duplicated clusters of non‐TIR‐like NBS‐LRR‐encoding genes on sunflower chromosomes 1 and 13. Theor. Appl. Genet. 122, 1211–1221. 21293840 10.1007/s00122-010-1525-0

[tpj14157-bib-0005] Badouin, H. , Gouzy, J. , Grassa, C.J. ***et al.*** (2017) The sunflower genome provides insights into oil metabolism, flowering and Asterid evolution. Nature, 546, 148. 28538728 10.1038/nature22380

[tpj14157-bib-0006] Bhattacharjee, S. , Hiller, N.L. , Liolios, K. , Win, J. , Kanneganti, T.D. , Young, C. , Kamoun, S. and Haldar, K. (2006) The malarial host‐targeting signal is conserved in the Irish potato famine pathogen. PLoS Pathog. 2, 453–465. 10.1371/journal.ppat.0020050 PMC1464399 16733545

[tpj14157-bib-0007] Bos, J.I.B. , Kanneganti, T.‐D. , Young, C. , Cakir, C. , Huitema, E. , Win, J. , Armstrong, M.R. , Birch, P.R.J. and Kamoun, S. (2006) The C‐terminal half of Phytophthora infestans RXLR effector AVR3a is sufficient to trigger R3a‐mediated hypersensitivity and suppress INF1‐induced cell death in *Nicotiana benthamiana* . Plant J. 48, 165–176. 16965554 10.1111/j.1365-313X.2006.02866.x

[tpj14157-bib-0008] Boutemy, L.S. , King, S.R.F. , Win, J. , Hughes, R.K. , Clarke, T.A. , Blumenschein, T.M.A. , Kamoun, S. and Banfield, M.J. (2011) Structures of Phytophthora RXLR effector proteins _ A conserved but adaptable fold underpins functional diversity. J. Biol. Chem. 286, 35834–35842. 21813644 10.1074/jbc.M111.262303 PMC3195559

[tpj14157-bib-0009] Burrus, M. , Molinier, J. , Himber, C. , Hunold, R. , Bronner, R. , Rousselin, P. and Hahne, G. (1996) Agrobacterium‐mediated transformation of sunflower (*Helianthus annuus* L) shoot apices: transformation patterns. Mol. Breeding 2, 329–338.

[tpj14157-bib-0010] Caplan, J.L. , Kumar, A.S. , Park, E. , Padmanabhan, M.S. , Hoban, K. , Modla, S. , Czymmek, K. and Dinesh‐Kumar, S.P. (2015) Chloroplast stromules function during innate immunity. Dev. Cell 34, 45–57. 26120031 10.1016/j.devcel.2015.05.011 PMC4596411

[tpj14157-bib-0011] Cock, P.J.A. and Pritchard, L . (2014) Galaxy as a platform for identifying candidate pathogen effectors In Plant‐Pathogen Interactions: Methods and Protocols, 2nd Edition (Birch P., Jones J.T. and Bos J.I.B., eds). Totowa: Humana Press Inc, pp. 3–15. 10.1007/978-1-62703-986-4_1 24643548

[tpj14157-bib-0012] Cooke, D.E.L. , Cano, L.M. , Raffaele, S. ***et al.*** (2012) Genome analyses of an aggressive and invasive lineage of the Irish potato famine pathogen. PLoS Pathog. 8, e1002940. 23055926 10.1371/journal.ppat.1002940 PMC3464212

[tpj14157-bib-0013] Dangl, J.L. , Horvath, D.M. and Staskawicz, B.J. (2013) Pivoting the plant immune system from dissection to deployment. Science 341, 746–751. 23950531 10.1126/science.1236011 PMC3869199

[tpj14157-bib-0014] Deslandes, L. and Genin, S. (2014) Opening the *Ralstonia solanacearum* type III effector tool box: insights into host cell subversion mechanisms. Curr. Opin. Plant Biol. 20, 110–117. 24880553 10.1016/j.pbi.2014.05.002

[tpj14157-bib-0015] Dussert, Y. , Gouzy, J. , Richart‐Cervera, S. , Mazet, I.D. , Delière, L. , Couture, C. , Legrand, L. , Piron, M.‐C. , Mestre, P. and Delmotte, F. (2016) Draft genome sequence of *Plasmopara viticola*, the grapevine downy mildew pathogen. Genome Announc. 4, e00987–16. 27660780 10.1128/genomeA.00987-16 PMC5034131

[tpj14157-bib-0016] Dussert, Y. , Mazet, I.D. , Couture, C. , Gouzy, J. , Piron, M.‐C. , Rispe, C. , Mestre, P. and Delmotte, F. (2018) A high‐quality grapevine downy mildew genome assembly reveals rapidly evolving and lineage‐specific putative host adaptation genes. bioRxiv. 10.1101/350041. PMC6660063 30847481

[tpj14157-bib-0017] Essl, D. , Dirnberger, D. , Gomord, V. , Strasser, R. , Faye, L. , Glossl, J. and Steinkellner, H. (1999) The N‐terminal 77 amino acids from tobacco N‐acetylglucosaminyltransferase I are sufficient to retain a reporter protein in the Golgi apparatus of *Nicotiana benthamiana* cells. FEBS Lett. 453, 169–173. 10403396 10.1016/s0014-5793(99)00712-7

[tpj14157-bib-0018] Fawke, S. , Doumane, M. and Schornack, S. (2015) Oomycete interactions with plants: infection strategies and resistance principles. Microbiol. Mol. Biol. Rev. 79, 263–280. 26041933 10.1128/MMBR.00010-15 PMC4468149

[tpj14157-bib-0019] Franchel, J. , Bouzidi, M.F. , Bronner, G. , Vear, F. , Nicolas, P. and Mouzeyar, S. (2013) Positional cloning of a candidate gene for resistance to the sunflower downy mildew, *Plasmopara halstedii* race 300. Theor. Appl. Genet. 126, 359–367. 23052021 10.1007/s00122-012-1984-6

[tpj14157-bib-0020] Franz, M. , Lopes, C.T. , Huck, G. , Dong, Y. , Sumer, O. and Bader, G.D. (2016) Cytoscape.js: a graph theory library for visualisation and analysis. Bioinformatics, 32, 309–311. 26415722 10.1093/bioinformatics/btv557 PMC4708103

[tpj14157-bib-0021] Gascuel, Q. , Martinez, Y. , Boniface, M.C. , Vear, F. , Pichon, M. and Godiard, L. (2015) The sunflower downy mildew pathogen *Plasmopara halstedii* . Mol. Plant Pathol. 16, 109–122. 25476405 10.1111/mpp.12164 PMC6638465

[tpj14157-bib-0022] Gascuel, Q. , Bordat, A. , Sallet, E. , Pouilly, N. , Carrere, S. , Roux, F. , Vincourt, P. and Godiard, L. (2016a) Effector polymorphisms of the sunflower downy mildew pathogen *Plasmopara halstedii* and their use to identify pathotypes from field isolates. PLoS ONE, 11, e0148513. 26845339 10.1371/journal.pone.0148513 PMC4742249

[tpj14157-bib-0023] Gascuel, Q. , Buendia, L. , Pecrix, Y. , Blanchet, N. , Muños, S. , Vear, F. and Godiard, L. (2016b) RXLR and CRN effectors from the sunflower downy mildew pathogen *Plasmopara halstedii* induce hypersensitive‐like responses in resistant sunflower lines. Front. Plant Sci. 7, 1887. 28066456 10.3389/fpls.2016.01887 PMC5165252

[tpj14157-bib-0024] Giesbers, A.K.J. , Pelgrom, A.J.E. , Visser, R.G.F. , Niks, R.E. , Van den Ackerveken, G. and Jeuken, M.J.W. (2017) Effector‐mediated discovery of a novel resistance gene against *Bremia lactucae* in a nonhost lettuce species. New Phytol., 216, 915–926. 28833168 10.1111/nph.14741 PMC5656935

[tpj14157-bib-0025] de Givry, S. , Bouchez, M. , Chabrier, P. , Milan, D. and Schiex, T. (2005) CARTHAGENE: multipopulation integrated genetic and radiation hybrid mapping. Bioinformatics, 21, 1703–1704. 15598829 10.1093/bioinformatics/bti222

[tpj14157-bib-0026] Goldfarb, D.S. , Gariepy, J. , Schoolnik, G. and Kornberg, R.D. (1986) Synthetic peptides as nuclear‐localization signals. Nature, 322, 641–644. 3638500 10.1038/322641a0

[tpj14157-bib-0027] Gomez, J. , Garcia, L.J. , Salazar, G.A. ***et al.*** (2013) BioJS: an open source JavaScript framework for biological data visualization. Bioinformatics, 29, 1103–1104. 23435069 10.1093/bioinformatics/btt100 PMC3624812

[tpj14157-bib-0028] Gouzy, J. , Corpet, F. and Kahn, D. (1999) Whole genome protein domain analysis using a new method for domain clustering. Comput. Chem. 23, 333–340. 10404623 10.1016/s0097-8485(99)00011-x

[tpj14157-bib-0029] Jones, J.D.G. and Dangl, J.L. (2006) The plant immune system. Nature, 444, 323–329. 17108957 10.1038/nature05286

[tpj14157-bib-0030] Jones, P. , Binns, D. , Chang, H.Y. ***et al.*** (2014) InterProScan 5: genome‐scale protein function classification. Bioinformatics, 30, 1236–1240. 24451626 10.1093/bioinformatics/btu031 PMC3998142

[tpj14157-bib-0031] Jones, J.D.G. , Vance, R.E. and Dangl, J.L. (2016) Intracellular innate immune surveillance devices in plants and animals. Science, 354, 6. 10.1126/science.aaf6395 27934708

[tpj14157-bib-0032] Krzywinski, M. , Schein, J. , Birol, I. , Connors, J. , Gascoyne, R. , Horsman, D. , Jones, S.J. and Marra, M.A. (2009) Circos: an information aesthetic for comparative genomics. Genome Res., 19, 1639–1645. 19541911 10.1101/gr.092759.109 PMC2752132

[tpj14157-bib-0033] Love, M.I. , Huber, W. and Anders, S. (2014) Moderated estimation of fold change and dispersion for RNA‐seq data with DESeq2. Genome Biol. 15, 550. 25516281 10.1186/s13059-014-0550-8 PMC4302049

[tpj14157-bib-0034] Ma, G.J. , Markell, S.G. , Song, Q.J. and Qi, L.L. (2017) Genotyping‐by‐sequencing targeting of a novel downy mildew resistance gene Pl (20) from wild *Helianthus argophyllus* for sunflower (*Helianthus annuus* L.). Theor. Appl. Genet. 130, 1519–1529. 28432412 10.1007/s00122-017-2906-4

[tpj14157-bib-0035] Mangin, B. , Pouilly, N. , Boniface, M.C. , Langlade, N.B. , Vincourt, P. , Vear, F. and Muños, S. (2017) Molecular diversity of sunflower populations maintained as genetic resources is affected by multiplication processes and breeding for major traits. Theor. Appl. Genet. 130, 1099–1112. 28255669 10.1007/s00122-017-2872-x

[tpj14157-bib-0036] Mestre, P. , Carrere, S. , Gouzy, J. , Piron, M.C. , de Labrouhe, D.T. , Vincourt, P. , Delmotte, F. and Godiard, L. (2016) Comparative analysis of expressed CRN and RXLR effectors from two Plasmopara species causing grapevine and sunflower downy mildew. Plant Pathol. 65, 767–781.

[tpj14157-bib-0037] Nielsen, H. (2017) Predicting secretory proteins with SignalP. Methods Mol. Biol. 1611, 59–73. 28451972 10.1007/978-1-4939-7015-5_6

[tpj14157-bib-0038] Ono, K. , Demchak, B. and Ideker, T. (2014) Cytoscape tools for the web age: D3.js and Cytoscape.js exporters. F1000Research, 3, 143. 25520778 10.12688/f1000research.4510.1 PMC4264639

[tpj14157-bib-0039] Pais, M. , Win, J. , Yoshida, K. , Etherington, G.J. , Cano, L.M. , Raffaele, S. , Banfield, M.J. , Jones, A. , Kamoun, S. and Saunders, D.G.O. (2013) From pathogen genomes to host plant processes: the power of plant parasitic oomycetes. Genome Biol. 14, 10. 10.1186/gb-2013-14-6-211 PMC3706818 23809564

[tpj14157-bib-0040] Petre, B. , Saunders, D.G.O. , Sklenar, J. , Lorrain, C. , Krasileva, K.V. , Win, J. , Duplessis, S. and Kamoun, S. (2016) Heterologous expression screens in *Nicotiana benthamiana* identify a candidate effector of the wheat yellow rust pathogen that associates with processing bodies. PLoS ONE, 11, 16. 10.1371/journal.pone.0149035 PMC4749346 26863009

[tpj14157-bib-0041] Qi, L.L. , Long, Y.M. , Jan, C.C. , Ma, G.J. and Gulya, T.J. (2015) Pl(17) is a novel gene independent of known downy mildew resistance genes in the cultivated sunflower (*Helianthus annuus* L.). Theor. Appl. Genet. 128, 757–767. 25673143 10.1007/s00122-015-2470-8

[tpj14157-bib-0042] Qi, L.L. , Foley, M.E. , Cai, X.W. and Gulya, T.J. (2016) Genetics and mapping of a novel downy mildew resistance gene, Pl (18), introgressed from wild *Helianthus argophyllus* into cultivated sunflower (*Helianthus annuus* L.). Theor. Appl. Genet. 129, 741–752. 26747047 10.1007/s00122-015-2662-2

[tpj14157-bib-0043] Radwan, O. , Bouzidi, M.F. , Vear, F. , Philippon, J. , de Labrouhe, D.T. , Nicolas, P. and Mouzeyar, S. (2003) Identification of non‐TIR‐NBS‐LRR markers linked to the Pl5/Pl8 locus for resistance to downy mildew in sunflower. Theor. Appl. Genet. 106, 1438–1446. 12750787 10.1007/s00122-003-1196-1

[tpj14157-bib-0044] Radwan, O. , Gandhi, S. , Heesacker, A. , Whitaker, B. , Taylor, C. , Plocik, A. , Kesseli, R. , Kozik, A. , Michelmore, R.W. and Knapp, S.J. (2008) Genetic diversity and genomic distribution of homologs encoding NBS‐LRR disease resistance proteins in sunflower. Mol. Genet. Genomics 280, 111–125. 18553106 10.1007/s00438-008-0346-1

[tpj14157-bib-0045] Rehmany, A.P. , Gordon, A. , Rose, L.E. , Allen, R.L. , Armstrong, M.R. , Whisson, S.C. , Kamoun, S. , Tyler, B.M. , Birch, P.R.J. and Beynon, J.L. (2005) Differential recognition of highly divergent downy mildew avirulence gene alleles by RPP1 resistance genes from two Arabidopsis lines. Plant Cell, 17, 1839–1850. 15894715 10.1105/tpc.105.031807 PMC1143081

[tpj14157-bib-0046] Saito, C. , Ueda, T. , Abe, H. , Wada, Y. , Kuroiwa, T. , Hisada, A. , Furuya, M. and Nakano, A. (2002) A complex and mobile structure forms a distinct subregion within the continuous vacuolar membrane in young cotyledons of Arabidopsis. Plant J. 29, 245–255. 11844103 10.1046/j.0960-7412.2001.01189.x

[tpj14157-bib-0047] Schornack, S. , van Damme, M. , Bozkurt, T.O. , Cano, L.M. , Smoker, M. , Thines, M. , Gaulin, E. , Kamoun, S. and Huitema, E. (2010) Ancient class of translocated oomycete effectors targets the host nucleus. Proc. Natl. Acad. Sci. USA 107, 17421–17426. 20847293 10.1073/pnas.1008491107 PMC2951462

[tpj14157-bib-0048] Shannon, P. , Markiel, A. , Ozier, O. , Baliga, N.S. , Wang, J.T. , Ramage, D. , Amin, N. , Schwikowski, B. and Ideker, T. (2003) Cytoscape: a software environment for integrated models of biomolecular interaction networks. Genome Res. 13, 2498–2504. 14597658 10.1101/gr.1239303 PMC403769

[tpj14157-bib-0049] Sharma, R. , Xia, X.J. , Cano, L.M. ***et al.*** (2015) Genome analyses of the sunflower pathogen *Plasmopara halstedii* provide insights into effector evolution in downy mildews and Phytophthora. BMC Genom. 16, 741. 10.1186/s12864-015-1904-7 PMC4594904 26438312

[tpj14157-bib-0050] Slabaugh, M.B. , Yu, J.K. , Tang, S.X. , Heesacker, A. , Hu, X. , Lu, G.H. , Bidney, D. , Han, F. and Knapp, S.J. (2003) Haplotyping and mapping a large cluster of downy mildew resistance gene candidates in sunflower using multilocus intron fragment length polymorphisms. Plant Biotechnol. J. 1, 167–185. 17156030 10.1046/j.1467-7652.2003.00016.x

[tpj14157-bib-0051] Vleeshouwers, V.G.A.A. , Rietman, H. , Krenek, P. ***et al.*** (2008) Effector genomics accelerates discovery and functional profiling of potato disease resistance and *Phytophthora infestans* avirulence genes. PLoS ONE, 3, e2875. 18682852 10.1371/journal.pone.0002875 PMC2483939

[tpj14157-bib-0052] Wang, Q. , Han, C. , Ferreira, A.O. ***et al.*** (2011) Transcriptional programming and functional interactions within the *Phytophthora sojae* RXLR effector repertoire. Plant Cell, 23, 2064–2086. 21653195 10.1105/tpc.111.086082 PMC3160037

[tpj14157-bib-0053] Waterhouse, R.M. , Seppey, M. , Simão, F.A. , Manni, M. , Ioannidis, P. , Klioutchnikov, G. , Kriventseva, E.V. and Zdobnov, E.M. (2018) BUSCO applications from quality assessments to gene prediction and phylogenomics. Mol. Biol. Evol. 35, 543–548. 29220515 10.1093/molbev/msx319 PMC5850278

[tpj14157-bib-0054] Whisson, S.C. , Boevink, P.C. , Moleleki, L. ***et al.*** (2007) A translocation signal for delivery of oomycete effector proteins into host plant cells. Nature, 450, 115–119. 17914356 10.1038/nature06203

[tpj14157-bib-0055] Win, J. , Krasileva, K.V. , Kamoun, S. , Shirasu, K. , Staskawicz, B.J. and Banfield, M.J. (2012) Sequence divergent RXLR effectors share a structural fold conserved across plant pathogenic oomycete species. PLoS Pathog. 8, e1002400. 22253591 10.1371/journal.ppat.1002400 PMC3257287

[tpj14157-bib-0056] Yin, L. , An, Y.H. , Qu, J.J. , Li, X.L. , Zhang, Y.L. , Dry, I. , Wu, H.J. and Lu, J. (2017) Genome sequence of *Plasmopara viticola* and insight into the pathogenic mechanism. Sci. Rep. 7, 46553. 28417959 10.1038/srep46553 PMC5394536

[tpj14157-bib-0057] Zabala, M.D.T. , Littlejohn, G. , Jayaraman, S. ***et al.*** (2015) Chloroplasts play a central role in plant defence and are targeted by pathogen effectors. Nat Plants 1, 10. 10.1038/nplants.2015.74 27250009

[tpj14157-bib-0058] Zhang, Z.W. , Ma, G.J. , Zhao, J. , Markell, S.G. and Qi, L.L. (2017) Discovery and introgression of the wild sunflower‐derived novel downy mildew resistance gene Pl (19) in confection sunflower (*Helianthus annuus* L.). Theor. Appl. Genet. 130, 29–39. 27677630 10.1007/s00122-016-2786-z

