# Supplementary material for: Sunflower resistance to multiple downy mildew pathotypes revealed by recognition of conserved effectors of the oomycete Plasmopara halstedii
Source: Plant J. 2019 Jan 7;97(4):730–48. doi: 10.1111/tpj.14157 (PMC6849628; doi:10.1111/tpj.14157)
Supplement: Supplementary file 1 — Figure S1. Plasmopara halstedii RXLR selection workflow and RXLR number detected by the different methods used. [file TPJ-97-730-s001.pdf]

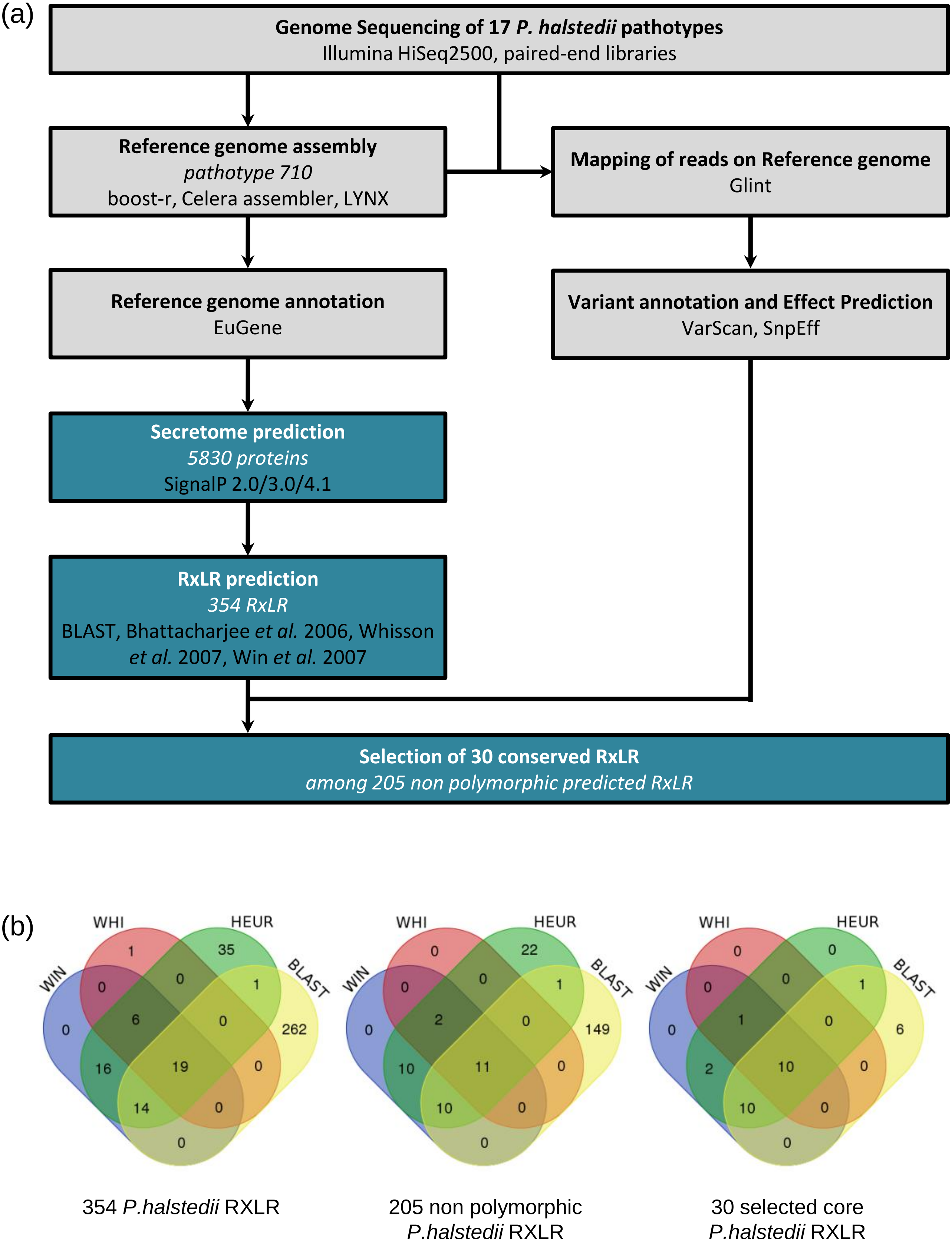

Figure S1. (a) *P halstedii* RXLR selection workflow. (b) Venn diagrams illustrating the number of *P halstedii* RXLR detected by the different methods used.
