# Supplementary material for: Sunflower resistance to multiple downy mildew pathotypes revealed by recognition of conserved effectors of the oomycete Plasmopara halstedii
Source: Plant J. 2019 Jan 7;97(4):730–48. doi: 10.1111/tpj.14157 (PMC6849628; doi:10.1111/tpj.14157)
Supplement: Supplementary file 3 — Figure S3. Online version of P. halstedii RXLR effector network* or pdf screenshots of the network images.*https://ianttoulouseinrafr/EFFECTOORES/webapp/data/clustering/#/ [file TPJ-97-730-s003.pdf]

Connected components numbers

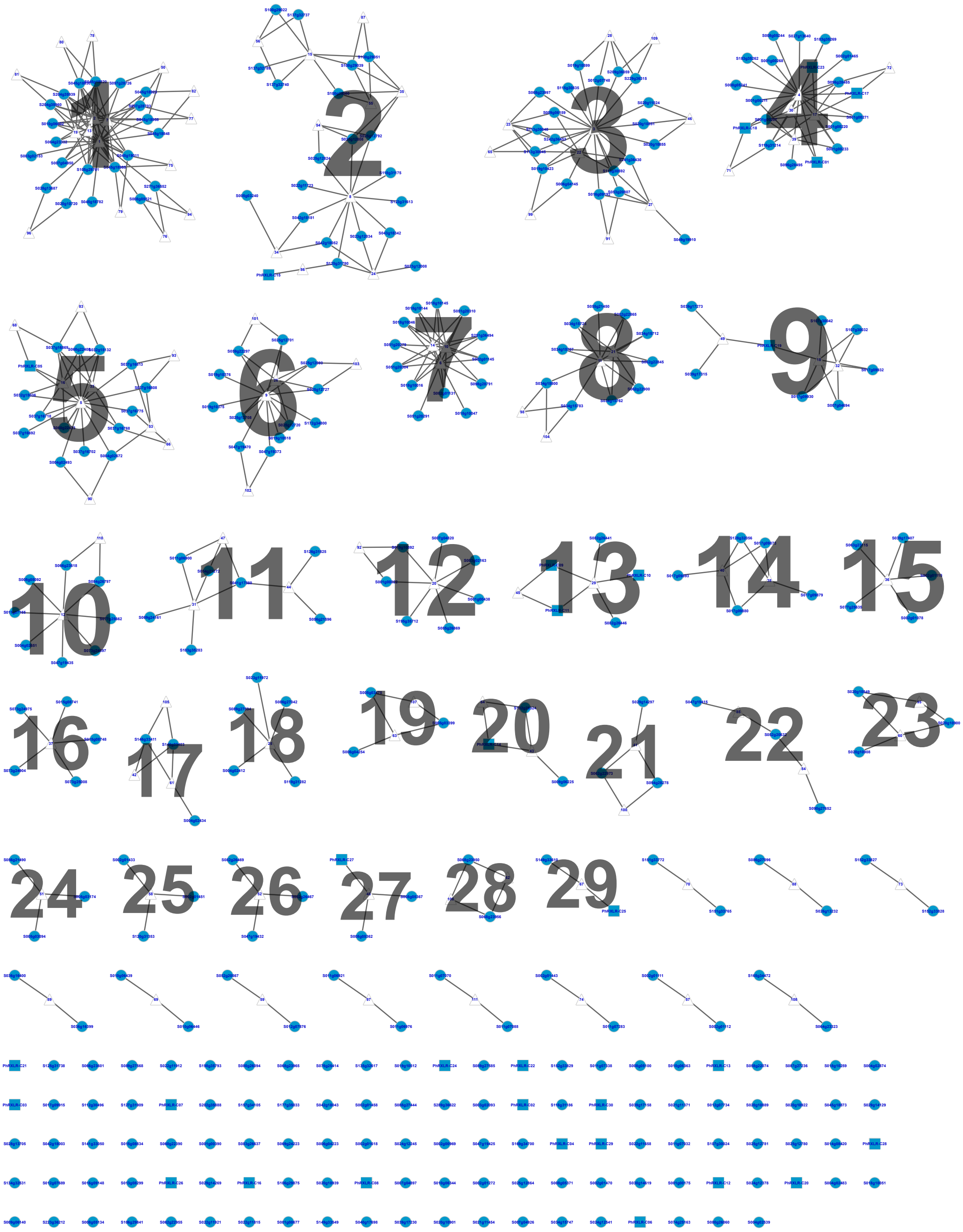

BlastP oomycete species

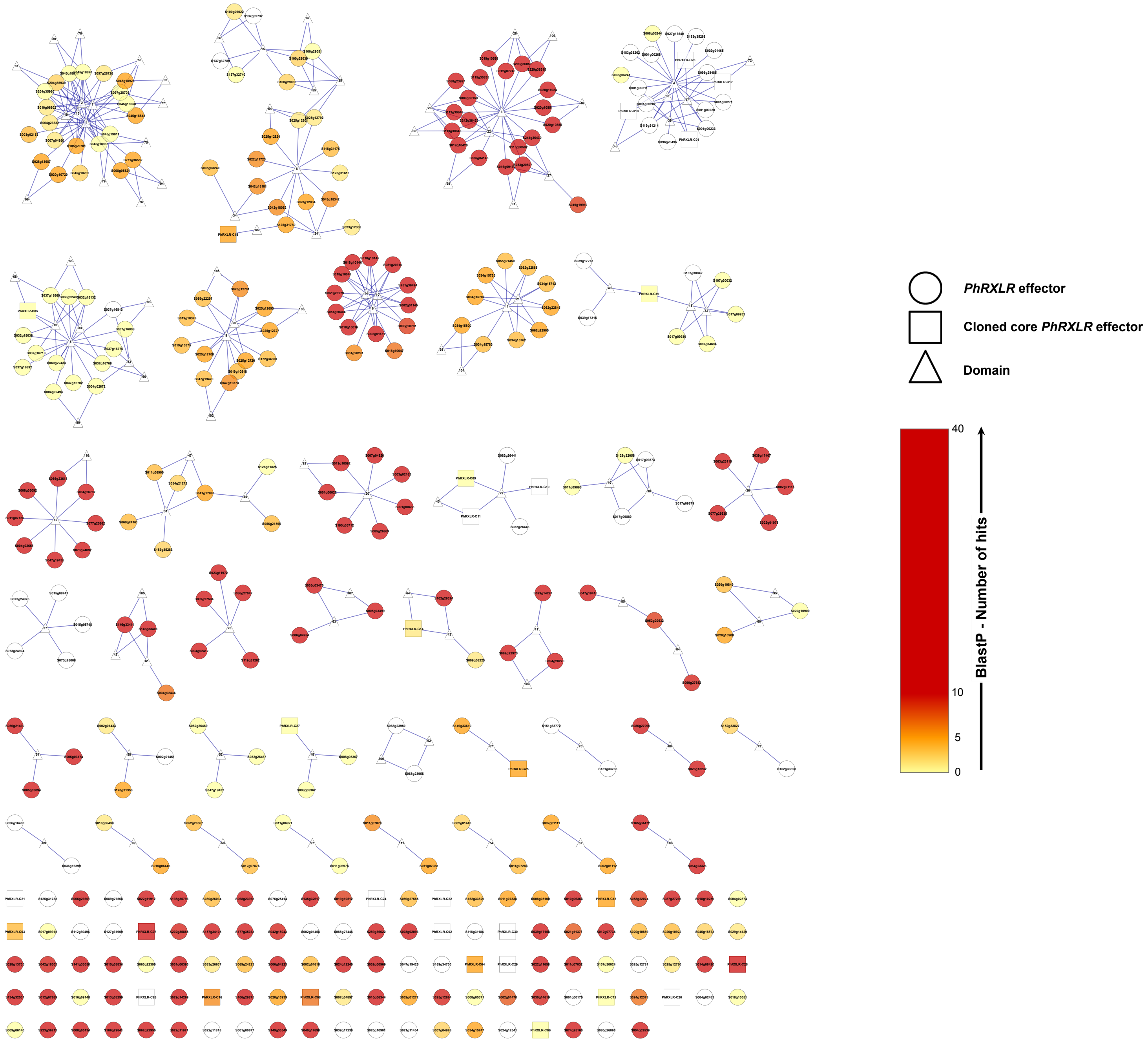

WY domains

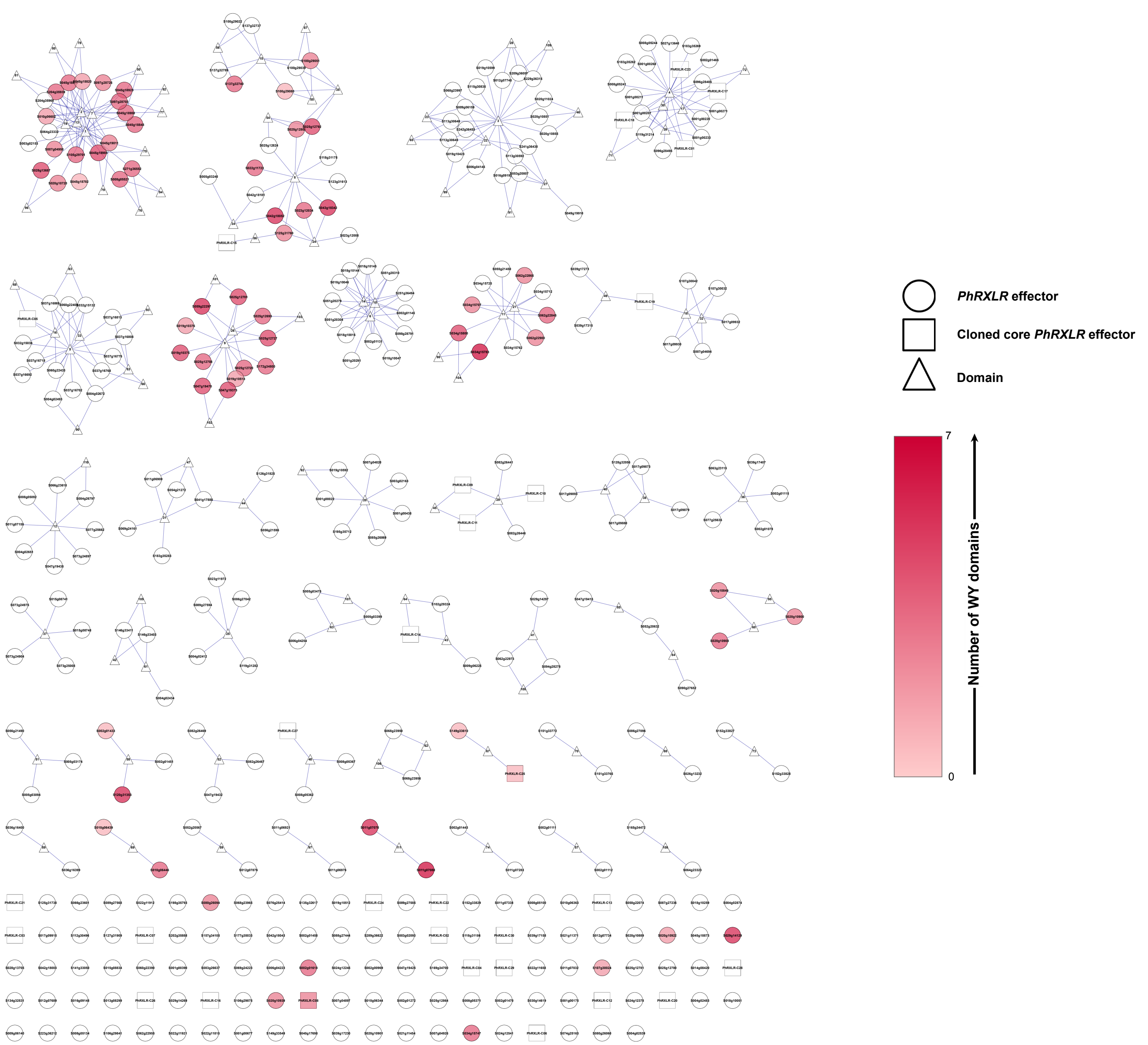
PhRXLR effector
Cloned core *PhRXLR* effector
Domain
7
0
Number of WY domains

P. halstedii pathotype polymorphism

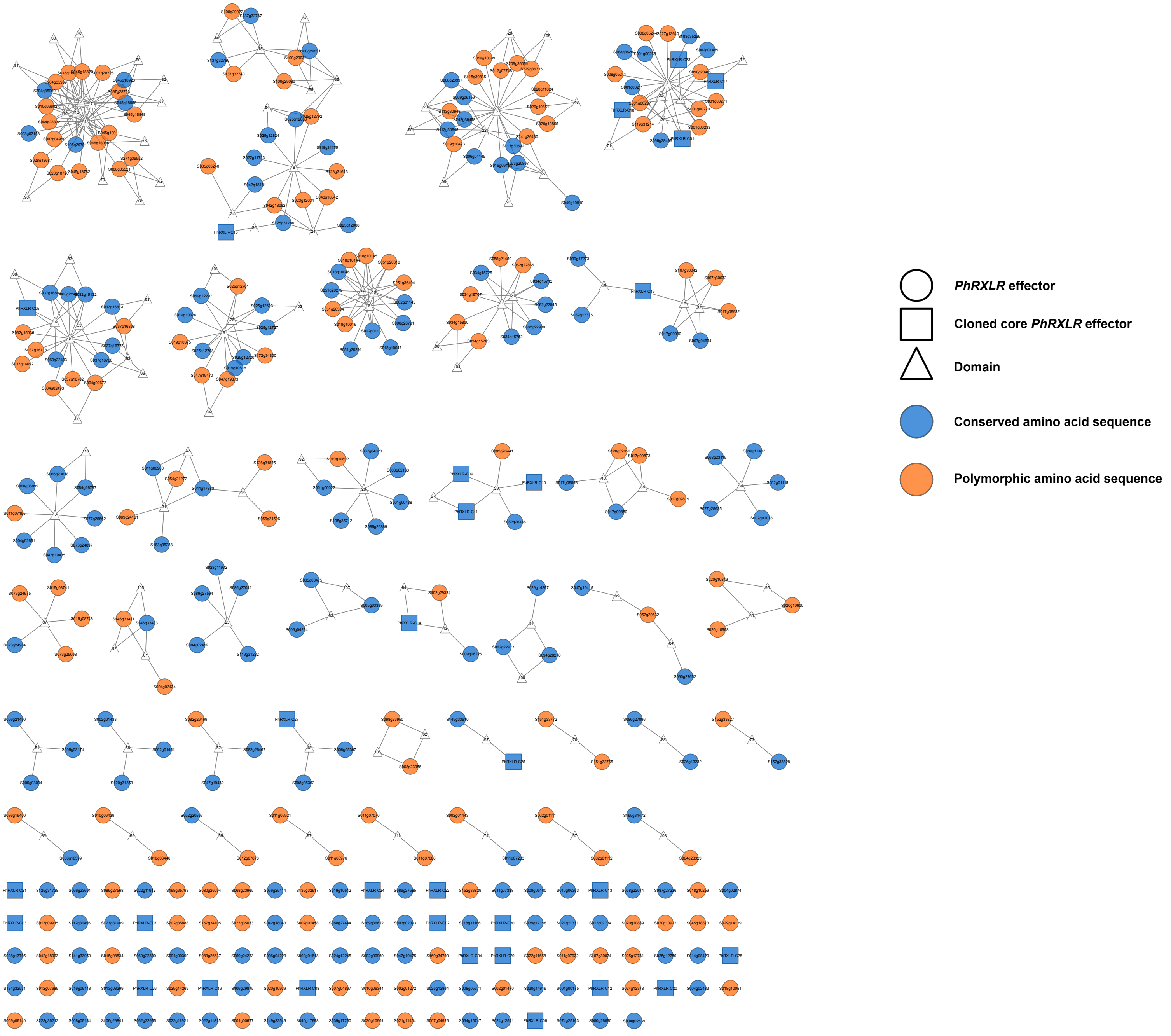

P. halstedii normalized gene expression in resistant sunflower 24hpi (R24h)

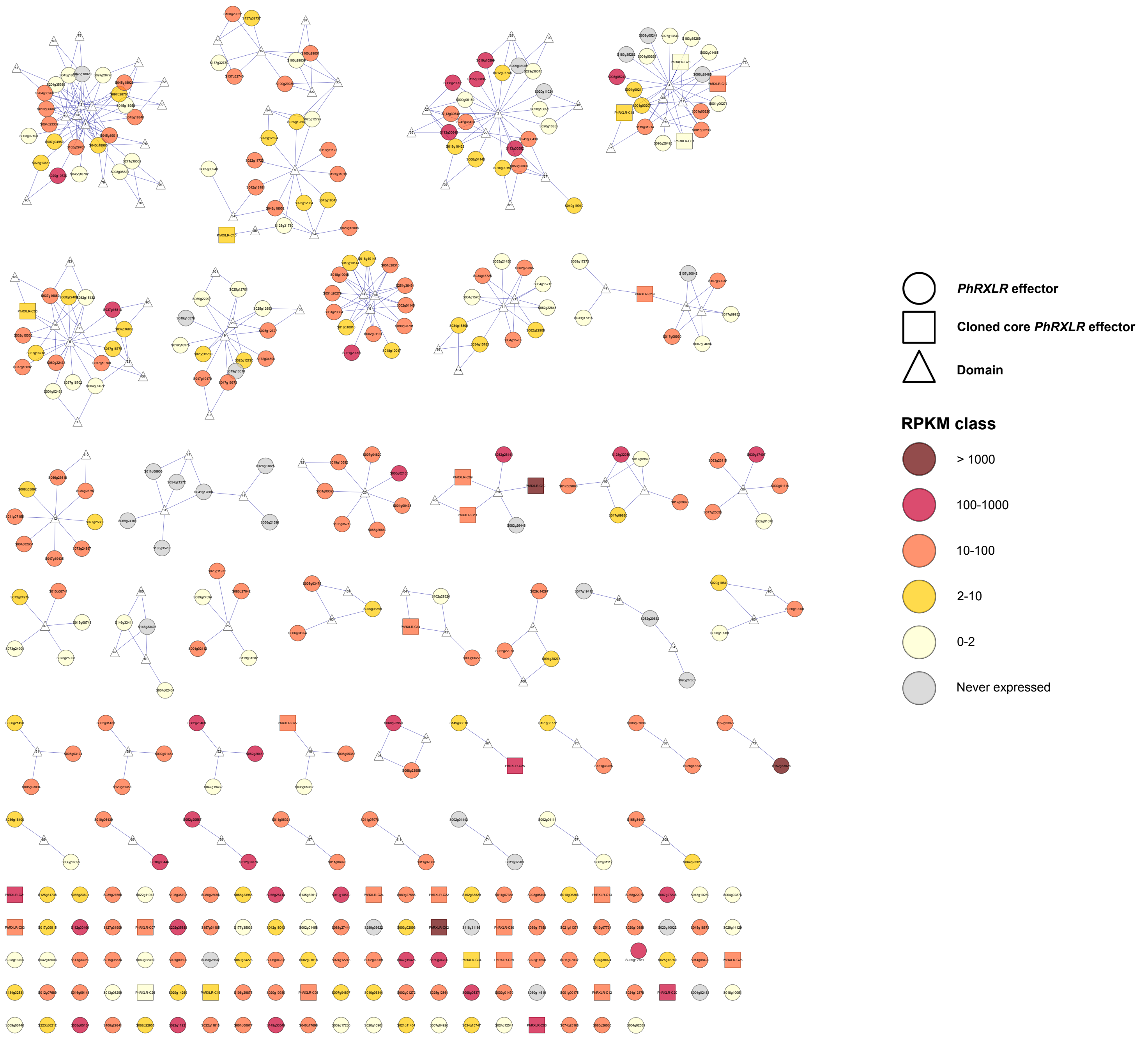

P. halstedii normalized gene expression in susceptible sunflower 24hpi (S24h)

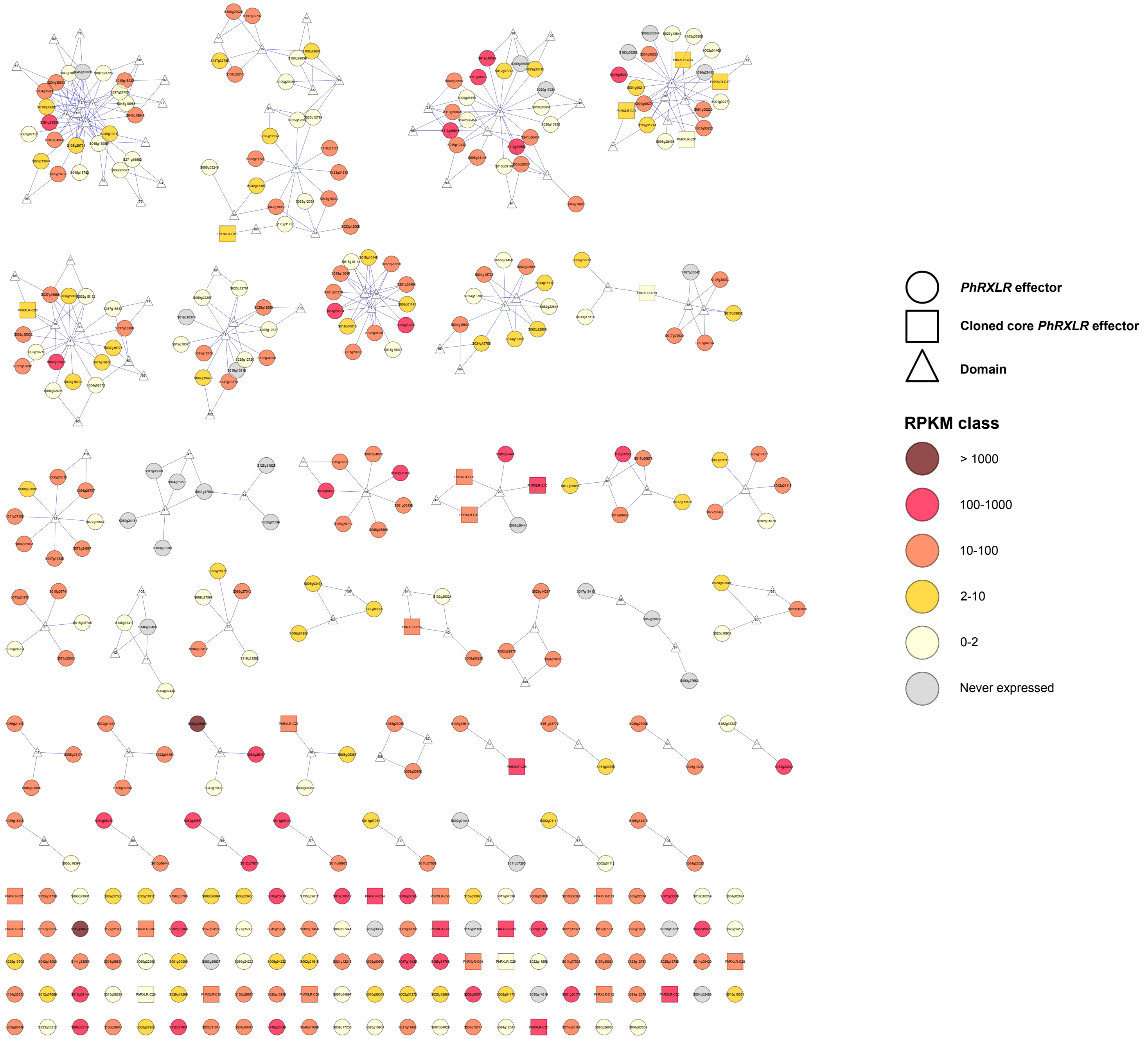

P. halstedii normalized gene expression in susceptible sunflower 10dpi (S10d)

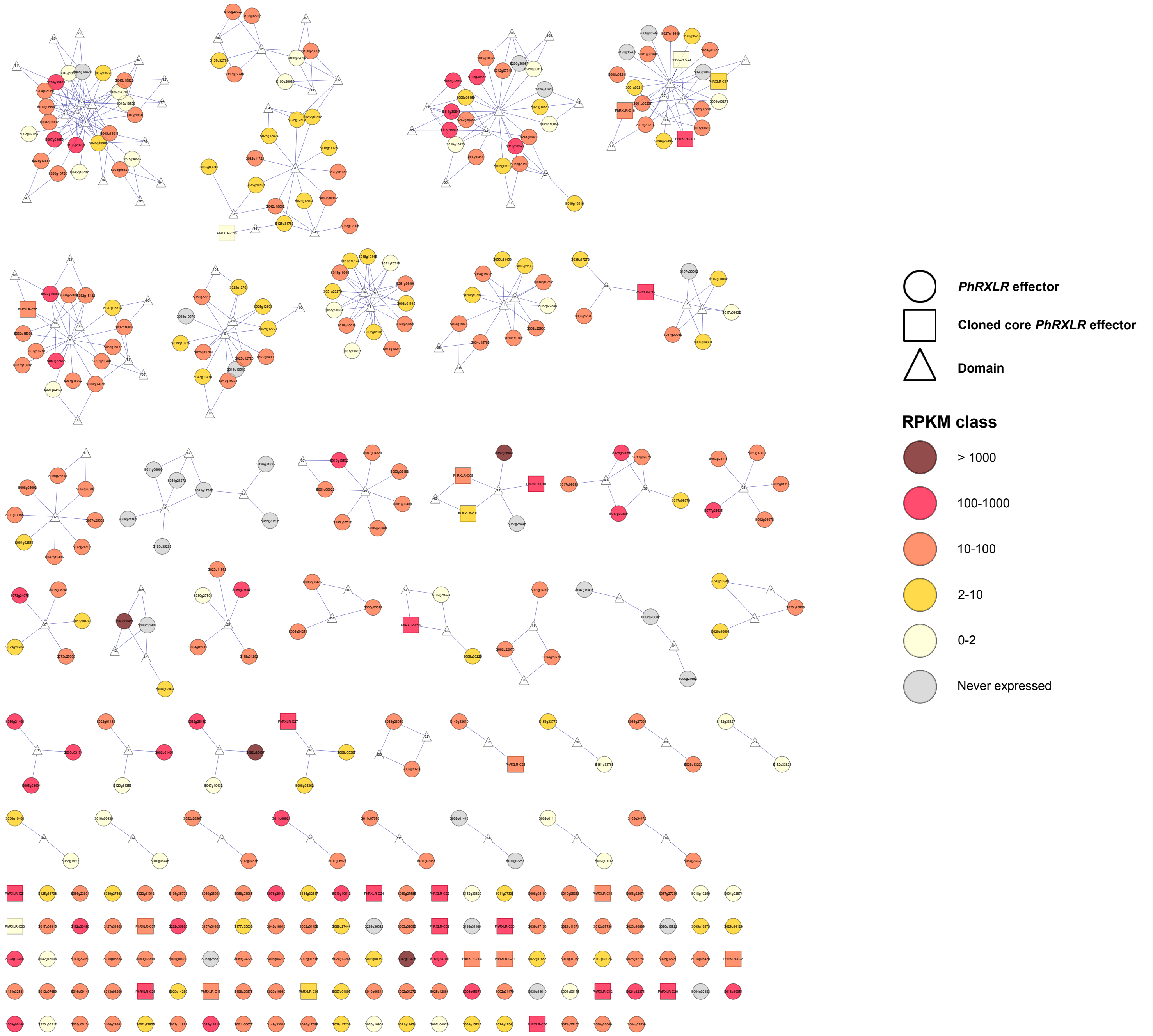

Fold Change R24h/S24h

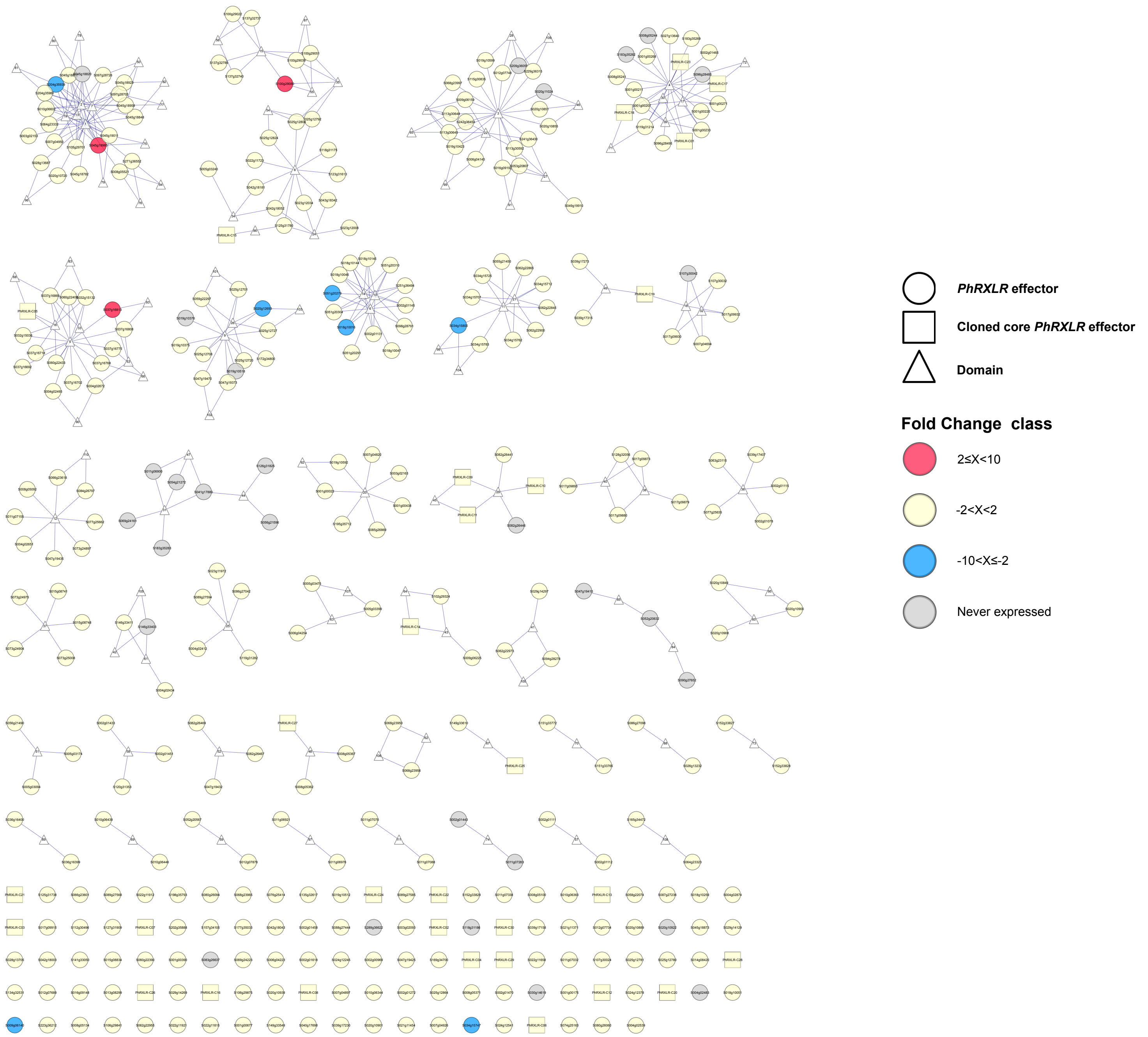

Fold Change S10d/S24h

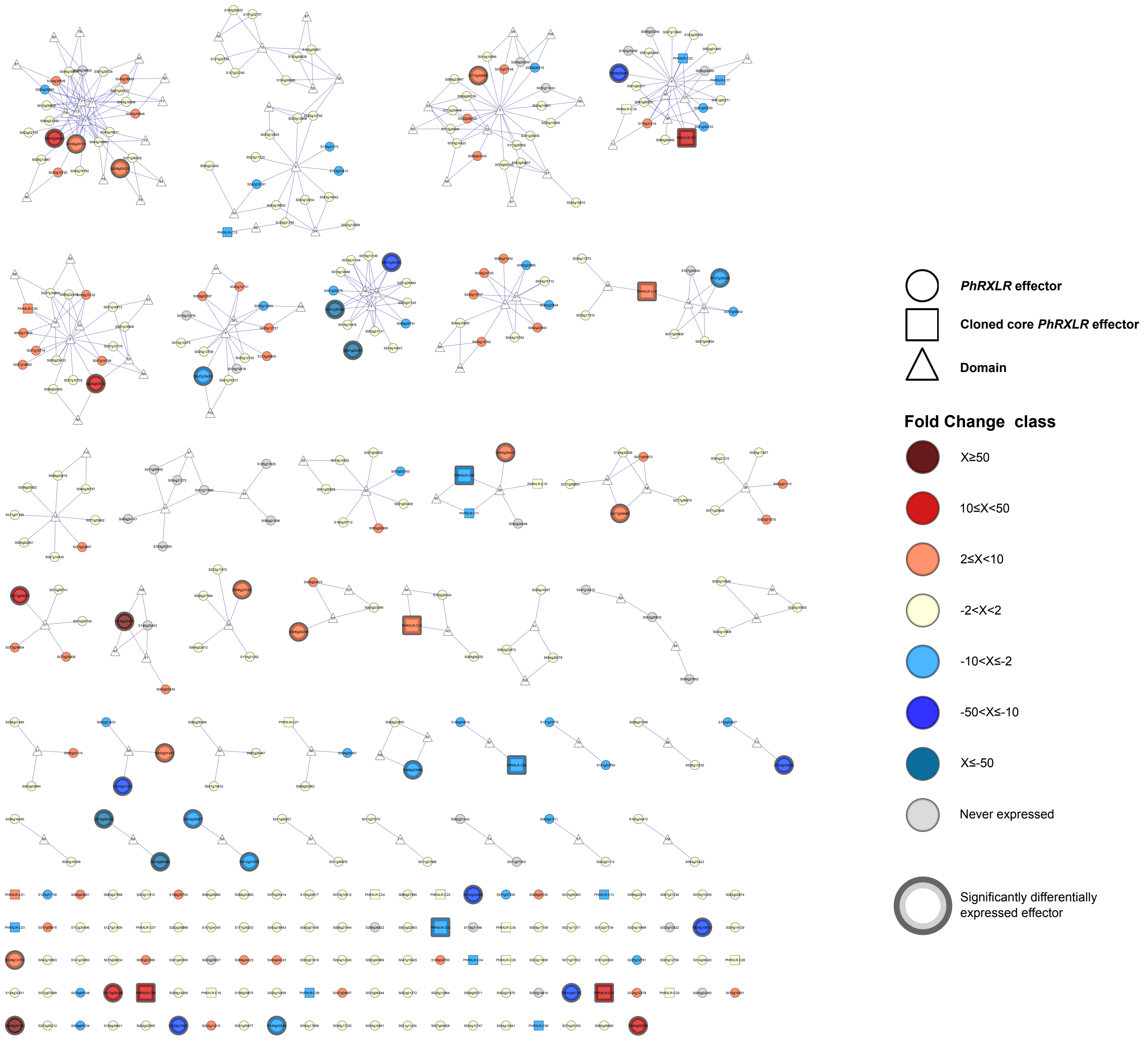

Figure S3. Online version of *Plasmopara halstedii* RXLR effector network or pdf screenshots of the different network images.
https://iant.toulouse.inra.fr/EFFECTOORES/webapp/data/clustering/#/
