# Supplementary material for: Sunflower resistance to multiple downy mildew pathotypes revealed by recognition of conserved effectors of the oomycete Plasmopara halstedii
Source: Plant J. 2019 Jan 7;97(4):730–48. doi: 10.1111/tpj.14157 (PMC6849628; doi:10.1111/tpj.14157)
Supplement: Supplementary file 4 — Figure S4. Expression analysis by RT‐qPCR of 30 P. halstedii core RXLR effectors in spores and during sunflower infection. [file TPJ-97-730-s004.pdf]

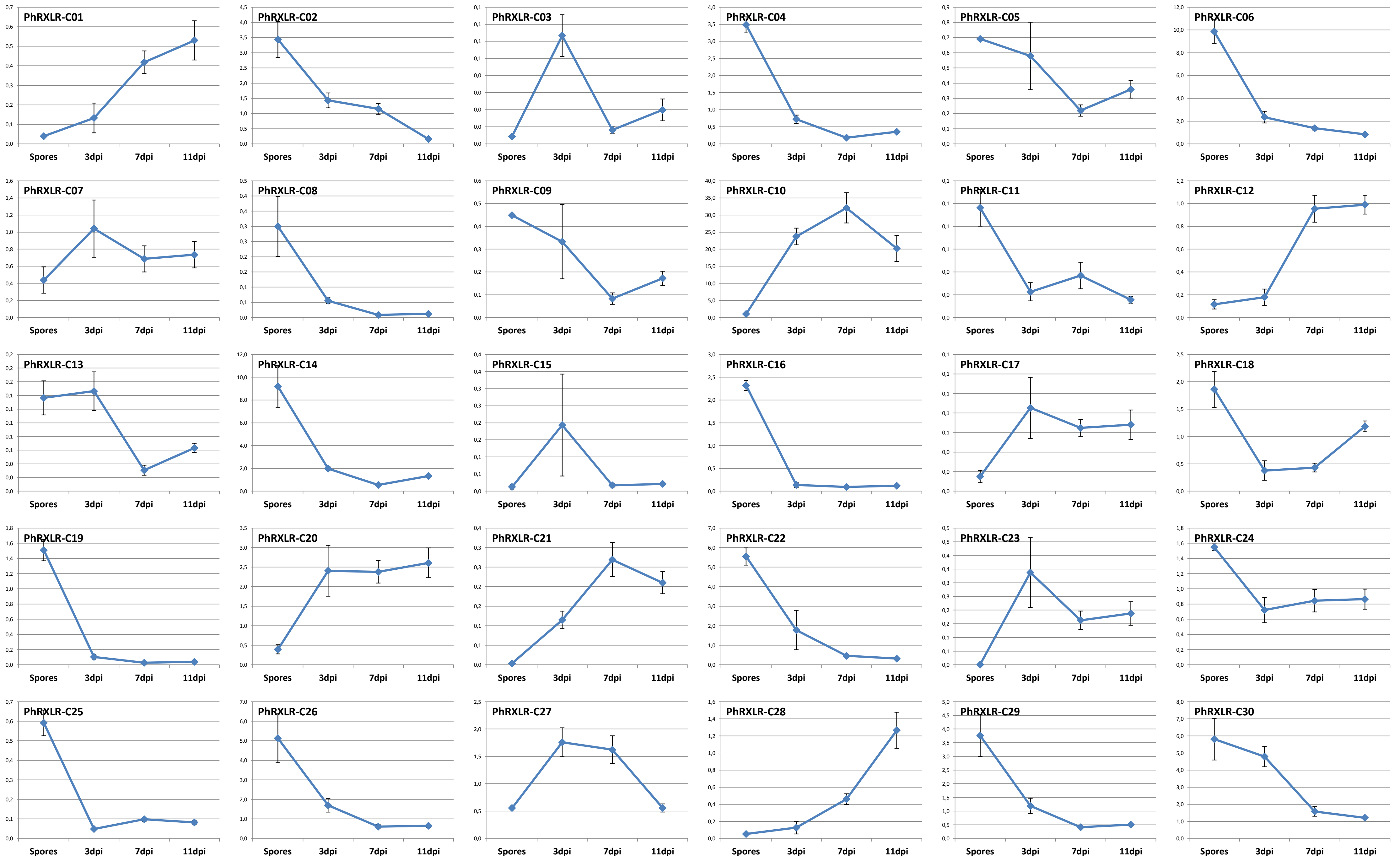

Figure S4. Expression analysis of 30 core *Plasmopara halstedii* RXLR effectors by RT-qPCR in *P. halstedii* (pathotype 710) spores and during sunflower infection. Transcript levels of core RXLR effectors during the infection of sunflower by *P. halstedii* at four time points (spores, 3, 7 and 11 dpi) were measured by RT-qPCR and normalized to PhRIBS3A (Gascuel *et al.*, 2016b). Error bars represent means ± s.d of three biological replicates.
