# Supplementary material for: Sunflower resistance to multiple downy mildew pathotypes revealed by recognition of conserved effectors of the oomycete Plasmopara halstedii
Source: Plant J. 2019 Jan 7;97(4):730–48. doi: 10.1111/tpj.14157 (PMC6849628; doi:10.1111/tpj.14157)
Supplement: Supplementary file 5 — Figure S5. Subcellular localizations of the 30 P. halstedii core RXLR effectors in sunflower cells. [file TPJ-97-730-s005.pdf]

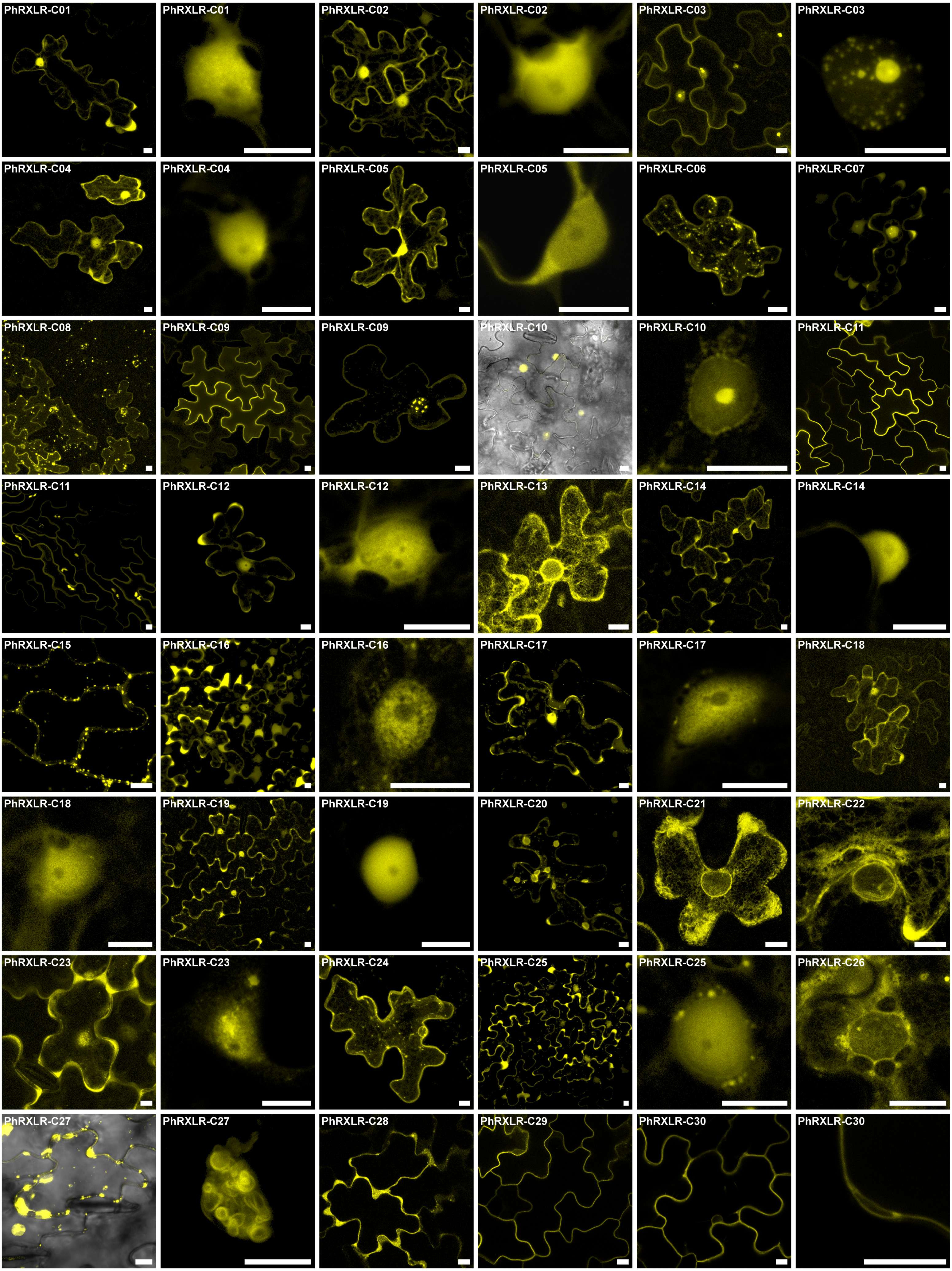

Fig. S5 Subcellular localizations of the 30 *P. halstedii* core RXLR effectors in sunflower cells.
Confocal images of p35S-YFP-PhRXLR constructs that were transiently expressed in sunflower leaves by agroinfiltration. Scale bar, 10 µm.
See Table 1 for localizations.
