# Supplementary material for: Sunflower resistance to multiple downy mildew pathotypes revealed by recognition of conserved effectors of the oomycete Plasmopara halstedii
Source: Plant J. 2019 Jan 7;97(4):730–48. doi: 10.1111/tpj.14157 (PMC6849628; doi:10.1111/tpj.14157)
Supplement: Supplementary file 6 — Figure S6. Colocalization studies of YFP‐PhRXLR core effector constructs with RFP‐tagged markers. [file TPJ-97-730-s006.pdf]

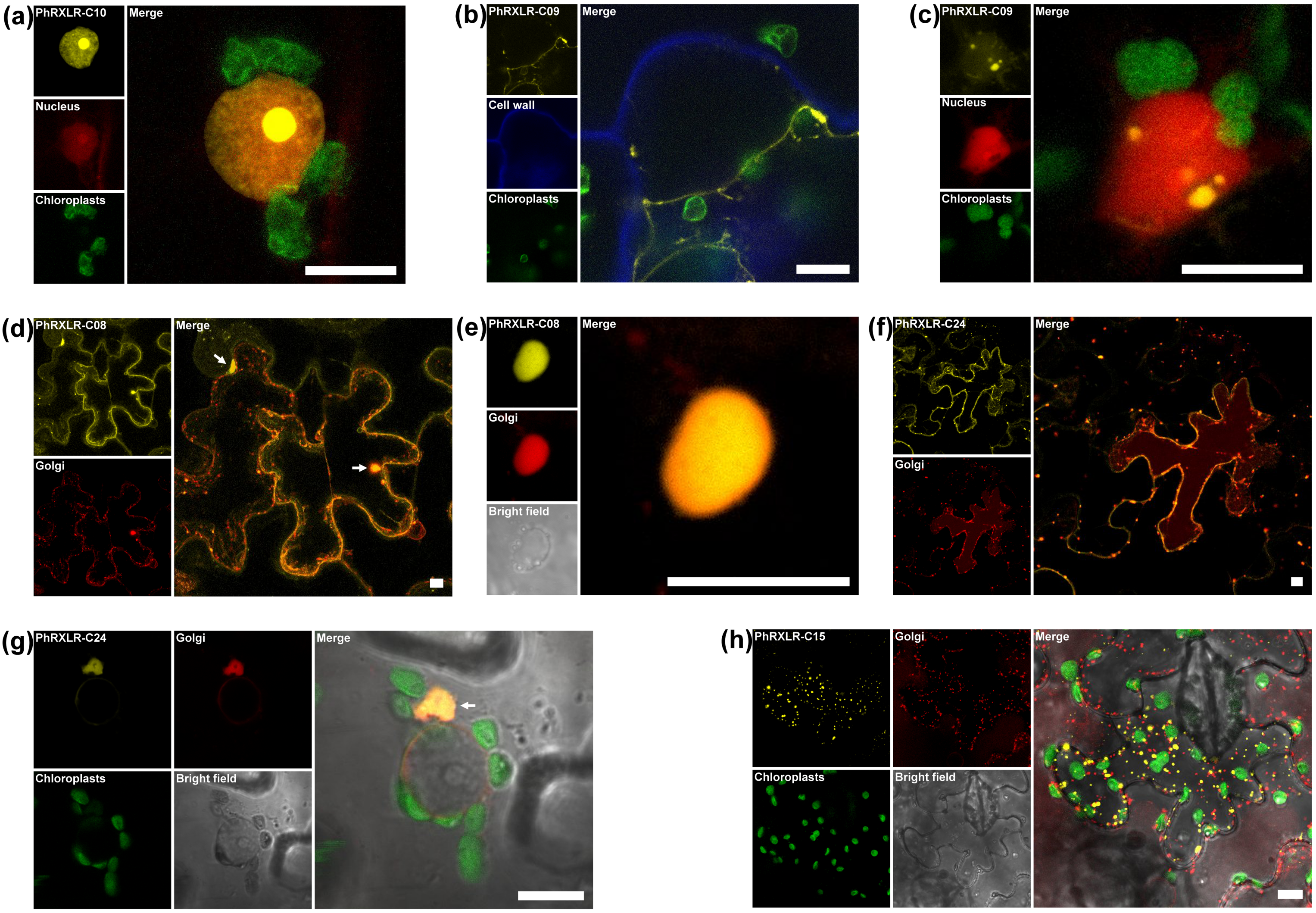

Fig. S6 Colocalization studies of YFP-PhRXLR core effector constructs with RFP-tagged markers.
Confocal images of p35S-YFP-PhRXLR and RFP-tagged marker constructs were transiently expressed by agroinfiltration in sunflower (a, c) and *N. benthamiana* leaves (b, d-h). The N-Acetyl Glucosaminyltransferase I from *Nicotiana tabacum* (Essl *et al.*, 1999) fused to RFP (GntI-RFP) was used as a marker of Golgi bodies, the large tumor antigen from SV40 (SVLT-RFP) was used as a specific nuclear marker (Goldfarb *et al.*, 1986). Chloroplasts were visualized by autofluorescence and cell walls by calcofluor staining. Scale bar, 10 µm.
(a) YFP-PhRXLR-C10 targets the nucleus and the nucleolus labeled with SVLT-RFP. (b) YFP-PhRXLR-C09 targets plasma membrane in a plasmolyzed cell and (c) forms aggregates in the nucleus. (d, e) YFP-PhRXLR-C08 colocalizes with GntI-RFP in Golgi bodies and in suspected Processing bodies (P-bodies), indicated by arrows in (d); an enlarged view of a P-body like structure (e) shows a local accumulation of both YFP-PhRXLR-C08 and GntI-RFP fusion proteins. (f, g) same observations as (d) and (e) for YFP-PhRXLR-C24. (h) YFP-PhRXLR-C15 showed a signal in undefined small bodies of variable size clearly distinct from Golgi bodies.
