# Supplementary material for: Sunflower resistance to multiple downy mildew pathotypes revealed by recognition of conserved effectors of the oomycete Plasmopara halstedii
Source: Plant J. 2019 Jan 7;97(4):730–48. doi: 10.1111/tpj.14157 (PMC6849628; doi:10.1111/tpj.14157)
Supplement: Supplementary file 7 — Figure S7. Recognition of P. halstedii RXLR core effectors in resistant sunflower lines. [file TPJ-97-730-s007.pdf]

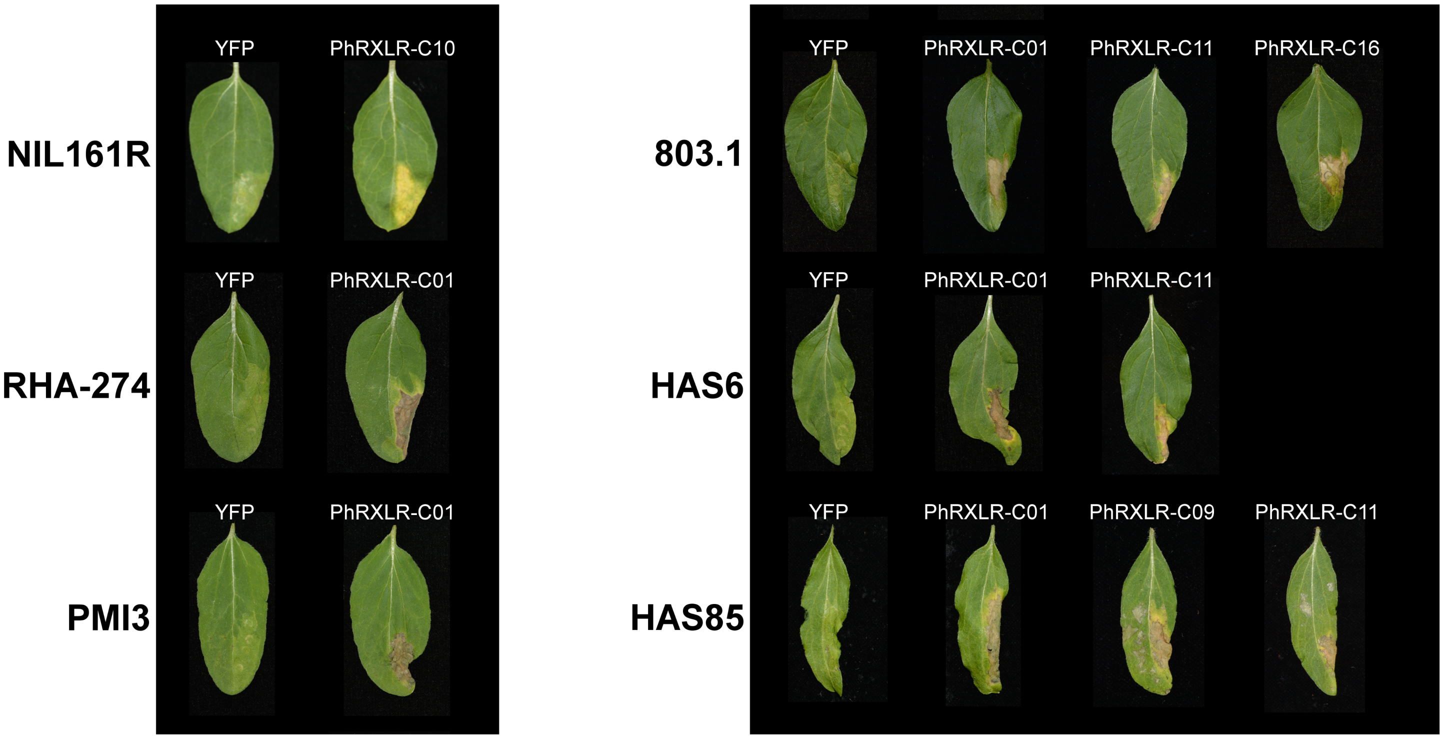

Fig. S7 Recognition of *P. halstedii* RXLR core effectors in resistant sunflower lines.
P. halstedii effectors induced either Hypersensitive Response (HR) cell death (PhRXLR-C01, -C11 and -C16) or a strong discoloration of the infiltrated leaf area in the line NIL161-R (PhRXLR-C10). PhRXLR-C01 induced an HR at 4 days post infiltration (dpil) in 3 differential lines RHA-274, PMI3 and 803-1. PhRXLR-C11 and PhRXLR-C16 both induced HR cell death in the differential line 803-1 at 6 dpil. The 2 lines, HAS6 and HAS85, carrying as yet unpublished resistances responded by an HR at 7 dpil to PhRXLR-C01 and PhRXLR-C11 effectors, but HAS85 revealed a novel HR-inducing effector, PhRXLR-C09.
