# Supplementary material for: Sunflower resistance to multiple downy mildew pathotypes revealed by recognition of conserved effectors of the oomycete Plasmopara halstedii
Source: Plant J. 2019 Jan 7;97(4):730–48. doi: 10.1111/tpj.14157 (PMC6849628; doi:10.1111/tpj.14157)
Supplement: Supplementary file 8 — Figure S8. Subfunctionalisation of the P. halstedii RXLR family of Connected Component 13. [file TPJ-97-730-s008.pdf]

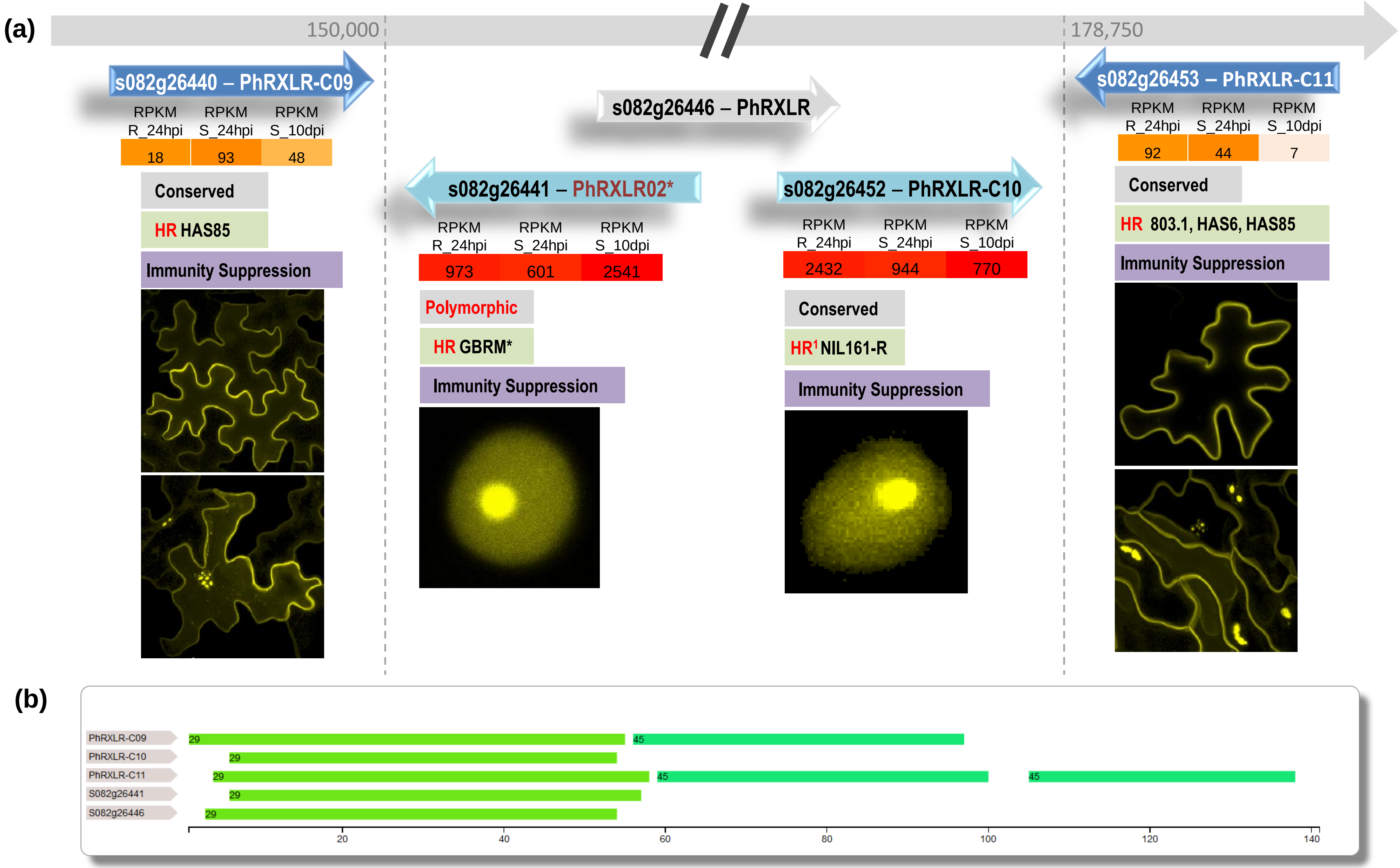

Figure S8. Subfunctionalisation of a *Plasmopara halstedii* RXLR family of Connected Component 13

(a) The 5 PhRXLR effector genes of CC13 are physically close on the same genomic scaffold indicated on top of the figure and probably resulted from duplication events. PhRXLR-C10 showed nuclear and nucleolar localization similar to the polymorphic effector PhRXLR02 (s082g26441) (Gascuel *et al.,* 2016a and 2016b), while both PhRXLR-C09 and -C11 were targeted to the plasma membrane and nucleus (but not to the nucleolus). The fact that these related effectors are recognized by different resistances in sunflower and are localized differently might reflect subfunctionalization events in order to create variant forms of effectors, escaping plant recognition. *Gascuel Q. *et al.*, 2016b.

(b) Organization of the 5 PhRXLR of CC13 in 2 different Mkdom2 domains 29 and 45.
