# Supplementary material for: Sunflower resistance to multiple downy mildew pathotypes revealed by recognition of conserved effectors of the oomycete Plasmopara halstedii
Source: Plant J. 2019 Jan 7;97(4):730–48. doi: 10.1111/tpj.14157 (PMC6849628; doi:10.1111/tpj.14157)
Supplement: Supplementary file 10 [file TPJ-97-730-s010.docx]

**SUPPORTING INFORMATION**

**Figure S1.** (a) *Plasmopara halstedii* RXLR selection workflow. (b) Venn diagrams illustrating the number of *P halstedii* RXLR detected by the different methods used.

**Figure S2.** Comparisons of *P. halstedii* genome assemblies (Sharma *et al.*, 2015, and this work) with three *Plasmopara viticola* genome assemblies obtained by Illumina (Dussert *et al*., 2016; Yin *et al*., 2017) and PacBio sequencing (Dussert *et al*., 2018).

(a) Number and total size of related scaffolds between *P. halstedii* and *P. viticola* genome assemblies. (b) Violin plots showing distribution of the *P. halstedii* scaffolds related to *P. viticola* scaffolds. Statistical significances between scaffolds distributions were evaluated by Wilcoxon signed ranks test (*, p<0.001). (c) Circos plots showing syntenic regions between one of the three largest scaffolds of each *P. viticola* assembly (in blue) and the *P. halstedii* related scaffolds (in black), for both *P. halstedii* genomes. Thicker bands correspond to syntenic regions larger than 10kb.

**Figure S3.** Online version of *P. halstedii* RXLR effector network* or pdf screenshots of the network images.

*[https://ianttoulouseinrafr/EFFECTOORES/webapp/data/clustering/#/](https://iant.toulouse.inra.fr/EFFECTOORES/webapp/data/clustering/#/)

**Figure S4.** Expression analysis by RT-qPCR of 30 *P. halstedii* core RXLR effectors in *P halstedii* (pathotype 710) spores and during sunflower infection.

Transcript levels of core RXLR effectors during the infection of sunflower by *P. halstedii* at four time points (spores, 3, 7 and 11 dpi) were measured by RT-qPCR and normalized to *PhRIBS3A* (Gascuel *et al.*, 2016b). Error bars represent means ± s.d of three biological replicates.

**Figure S5.** Subcellular localizations of the 30 YFP-PhRXLR core effector constructs in sunflower cells.

Confocal images of p35S-YFP-PhRXLR constructs that were transiently expressed in sunflower leaves by agroinfiltration. Scale bar, 10 µm. See Table 1 for localizations.

**Figure S6.** Colocalization studies of YFP-PhRXLR core effector constructs with RFP-tagged markers.

Confocal images of p35S-YFP-PhRXLR and RFP-tagged marker constructs were transiently expressed by agroinfiltration in sunflower (a, c) and *N benthamiana* leaves (b, d-h) The N-Acetyl Glucosaminyltransferase I from *Nicotiana tabacum* (Essl *et al.*, 1999) fused to RFP (GntI-RFP) was used as a marker of Golgi bodies, the large tumor antigen from SV40 (SVLT-RFP) was used as a specific nuclear marker (Goldfarb *et al.*, 1986). Chloroplasts were visualized by autofluorescence and cell walls by calcofluor staining. Scale bar, 10 µm.

(a) YFP-PhRXLR-C10 targets the nucleus and the nucleolus labeled with SVLT-RFP. (b) YFP-PhRXLR-C09 targets plasma membrane in a plasmolyzed cell and (c) forms aggregates in the nucleus. (d, e) YFP-PhRXLR-C08 colocalizes with GntI-RFP in Golgi bodies and in suspected Processing bodies (P-bodies), indicated by arrows in D; an enlarged view of a P-body like structure (e) shows a local accumulation of both YFP-PhRXLR-C08 and GntI-RFP fusion proteins. (f, g) same observations as d and e for YFP-PhRXLR-C24. (h) YFP-PhRXLR-C15 showed a signal in undefined small bodies of variable size clearly distinct from Golgi bodies.

**Figure S7.** Recognition of *P halstedii* RXLR core effectors in resistant sunflower lines.

*P halstedii* effectors induced either Hypersensitive Response (HR) cell death (PhRXLR-C01, -C11 and -C16) or a strong discoloration of the infiltrated leaf area in the line NIL161-R (PhRXLR-C10). PhRXLR-C01 induced an HR at 4 days post infiltration (dpil) in 3 differential lines RHA-274, PMI3 and 803-1. PhRXLR-C11 and PhRXLR-C16 both induced HR cell death in the differential line 803-1 at 6 dpil. The 2 lines, HAS6 and HAS85, carrying as yet unpublished resistances responded by an HR at 7 dpil to PhRXLR-C01 and PhRXLR-C11 effectors, but HAS85 revealed a novel HR-inducing effector, PhRXLR-C09.

**Figure S8.** Subfunctionalisation of the *Plasmopara halstedii* RXLR family of Connected Component 13.

(a) The 5 PhRXLR effector genes of CC13 are physically close on the same genomic scaffold indicated on top of the figure and probably resulted from duplication events. PhRXLR-C10 showed nuclear and nucleolar localization similar to the polymorphic effector PhRXLR02 (s082g26441) (Gascuel *et al.*, 2016a, Gascuel *et al.*, 2016b), while both PhRXLR-C09 and -C11 were targeted to the plasma membrane and nucleus (but not to the nucleolus). The fact that these related effectors are recognized by different resistances in sunflower and are localized differently might reflect subfunctionalization events in order to create variant forms of effectors, escaping plant recognition, * (Gascuel *et al.*, 2016b).

(b) Organization of the 5 PhRXLR of CC13 in 2 different Mkdom2 domains 29 and 45 (see Figure S2 for domains).

**Table S1.** Statistics, description and SRA accession numbers of raw genomic data of *Plasmopara halstedii* pathotype 710.

**Table S2.** Results of the *P. halstedii* 710 genome completeness analyses performed by BUSCO 3.0.2 with the database alveolata_stramenopiles_ensembl.

**Table S3.** Results of best reciprocal hit Blast between the *P. halstedii* 710 predicted proteins (this study) and the *P. halstedii* OS-Ph8-99-BlA4 predicted proteins (Sharma *et al.*, 2015).

**Table S4.** Description of the 354 *P halstedii* RXLR effectors with indication of the 30 core effectors selected for functional analysis, and their protein sequence in *P halstedii* pathotype 710.

**Table S5.** List of the Mkdom domains and Interpro hits for the 354 *P. halstedii* RXLRs.

**Table S6.** Statistics, description and SRA accession numbers of raw RNAseq data.

**Table S7.** RNAseq results for the 37 238 genes of *P halstedii* pathotype 710, obtained at 24h post inoculation in roots, both in resistant TSRM (R24h) and susceptible TS (S24h) sunflower genotypes (Gascuel *et al.*, 2015) and then at 10 dpi in hypocotyls of the susceptible TS line (S10d).

**Table S8.** Description of BlastP best hits of the 30 *P. halstedii* core RXLR effectors in oomycete species (nr 2017 01 10, E-value < 10^-4^).

**Table S9.** Sunflower candidate genes for *Pl22* resistance gene expressed upon *P. halstedii* infection.

**Table S10.** Sunflower lines used in this study.

**Table S11.** Taxonomy analysis of the *P. halstedii* 710 sequenced reads (from NCBI SRA).

**Table S12.** General features of *P. halstedii* 710 genomic scaffolds: GC percent, taxonomy relationships, Illumina reads coverage, intersection with RNAseq polyA data, similarity with Plasmopara public data.

**Table S13.** List of oomycetes and fungi used to construct the 2663 reference dataset of (putative) RXLRs for BlastP analyses on *P. halstedii* secretome.

**Table S14.** Primer sequences used for cloning and RT-qPCR experiments.

Gateway adaptator sequences (attB1 or attB2) were added in 5’ of each cloning primer.

**Table S15.** Mapping data file of PMI3xGH F3 progenies used for genotyping Pl22 resistance.

**Table S16.** Mapping data file of PMI3xIDAHO F3 progenies used for genotyping Pl22 resistance.
